# RcSRR1 interferes with the RcCSN5B-mediated deneddylation of RcCRL4 to modulate RcCO proteolysis and prevent rose flowering under red light

**DOI:** 10.1093/hr/uhaf025

**Published:** 2025-01-21

**Authors:** Weinan Wang, Jingjing Sun, Chunguo Fan, Guozhen Yuan, Rui Zhou, Jun Lu, Jinyi Liu, Changquan Wang

**Affiliations:** College of Horticulture, Nanjing Agricultural University, Nanjing 210095, China; Key Laboratory of Landscaping, Ministry of Agriculture and Rural Affairs, Nanjing 210095, China; Key Laboratory of State Forestry and Grassland Administration on Biology of Ornamental Plants in East China, Nanjing 210095, China; School of Civil Engineering, Yantai University, Yantai 264005, China; College of Horticulture, Nanjing Agricultural University, Nanjing 210095, China; Key Laboratory of Landscaping, Ministry of Agriculture and Rural Affairs, Nanjing 210095, China; Key Laboratory of State Forestry and Grassland Administration on Biology of Ornamental Plants in East China, Nanjing 210095, China; College of Horticulture, Nanjing Agricultural University, Nanjing 210095, China; Key Laboratory of Landscaping, Ministry of Agriculture and Rural Affairs, Nanjing 210095, China; Key Laboratory of State Forestry and Grassland Administration on Biology of Ornamental Plants in East China, Nanjing 210095, China; College of Horticulture, Nanjing Agricultural University, Nanjing 210095, China; Key Laboratory of Landscaping, Ministry of Agriculture and Rural Affairs, Nanjing 210095, China; Key Laboratory of State Forestry and Grassland Administration on Biology of Ornamental Plants in East China, Nanjing 210095, China; College of Horticulture, Nanjing Agricultural University, Nanjing 210095, China; Key Laboratory of Landscaping, Ministry of Agriculture and Rural Affairs, Nanjing 210095, China; Key Laboratory of State Forestry and Grassland Administration on Biology of Ornamental Plants in East China, Nanjing 210095, China; College of Horticulture, Nanjing Agricultural University, Nanjing 210095, China; Key Laboratory of Landscaping, Ministry of Agriculture and Rural Affairs, Nanjing 210095, China; Key Laboratory of State Forestry and Grassland Administration on Biology of Ornamental Plants in East China, Nanjing 210095, China; College of Horticulture, Nanjing Agricultural University, Nanjing 210095, China; Key Laboratory of Landscaping, Ministry of Agriculture and Rural Affairs, Nanjing 210095, China; Key Laboratory of State Forestry and Grassland Administration on Biology of Ornamental Plants in East China, Nanjing 210095, China; College of Horticulture, Nanjing Agricultural University, Nanjing 210095, China; Key Laboratory of Landscaping, Ministry of Agriculture and Rural Affairs, Nanjing 210095, China; Key Laboratory of State Forestry and Grassland Administration on Biology of Ornamental Plants in East China, Nanjing 210095, China

## Abstract

Light is essential for rose (*Rosa* spp.) growth and development. Different light qualities play differing roles in the rose floral transition, but the molecular mechanisms underlying their effects are not fully understood. Here, we observed that red light suppresses rose flowering and increases the expression of *sensitivity to red light reduced 1* (*RcSRR1*) compared with white light. Virus-induced gene silencing (VIGS) of *RcSRR1* led to early flowering under white light and especially under red light, suggesting that this gene is a flowering repressor with a predominant function under red light. We determined that RcSRR1 interacts with the COP9 signalosome subunit 5B (RcCSN5B), while RcCSN5B, RcCOP1, and RcCO physically interact with each other. Furthermore, the RcCSN5B-induced deneddylation of Cullin4-RING E3 ubiquitin ligase (RcCRL4) in rose was reduced by the addition of RcSRR1, suggesting that the interaction between RcSRR1 and RcCSN5B relieves the deneddylation of the RcCRL4-COP1/SPA complex to enhance RcCO proteolysis, which subsequently suppresses the transcriptional activation of *RcFT* and ultimately flowering. Far-red light-related sequence like 1 (RcFRSL3) was shown to specifically bind to the G-box motif of the *RcSRR1* promoter to repress its transcription, removing its inhibition of *RcFT* expression and inducing flowering. Red light inhibited *RcFRSL3* expression, thereby promoting the expression of *RcSRR1* to inhibit flowering. Taken together, these results provide a previously uncharacterized mechanism by which the RcFRSL3–RcSRR1–RcCSN5B module targets RcCO stability to regulate flowering under different light conditions in rose plants.

## Introduction

Roses (*Rosa* spp.) belong to the Rosaceae family and are one of the most popular cut flowers, with high symbolic value and cultural importance around the world [1–3]. Under favorable growth conditions, modern hybrid rose varieties can flower continuously from spring to autumn regardless of day-length, and are therefore generally considered to be a day-neutral (DN) woody species. A floral repressor *KSN* was reported to be responsible for the continuous flowering trait in rose [4–6]. In the continuous flowering rose variety ‘Old Blush’, a copia retrotransposon with a length of 10 kb was found to insert into the second intron of *KSN*, which blocks the expression of *KSN*, allowing it to flower regardless of day length [4]. Additional evidences suggest that the continuous flowering trait of rose is also influenced by other factors [7, 8].

Light is a major environmental signal that influences multiple aspects of plant growth and development. Light mainly affects plants in three dimensions: light intensity, photoperiod, and light quality [9–11]. Light quality (wavelength) is an important factor-impacting flowering. In roses, bud burst and meristem organogenesis are triggered by blue and red light, but inhibited by darkness and far-red light. Furthermore, red light is more effective at stimulating bud formation than blue light; at the end of the day shoots treated with low-intensity red light exhibit better plant growth and flower development than untreated shoots, although this can be reversed by far-red light [12–15]. The light-mediated regulation of flowering has been reported across a wide variety of plant species. Red light (high red:far-red ratio) has been shown to inhibit flowering in some LD plants, such as *Arabidopsis thaliana* [16], *Gypsophila paniculata* [17], *Eustoma grandiflorum* [18], *Campanula carpatica* [19], and *Matthiola incana* [20]. Plants use three main photoreceptors to perceive light quality: cryptochromes and phototropins sense blue/UV-A light, while phytochromes perceive red and far-red light. *Arabidopsis* has five distinct phytochromes, designated AtPHYA to AtPHYE, AtPHYB to AtPHYE have similar functions in the red-light response, with AtPHYB playing the dominant role, while AtPHYA is primarily responsible for the far-red light response. Red light promotes the degradation of CO by PHYB in a high expression of osmotically responsive genes1 (HOS1)-dependent and COP1-independent manner, which is crucial for inhibiting flowering [21–23]. Phytochrome-dependent late flowering (PHL) interacts with both CO and PHYB to protect CO from degradation by PHYB [24], while phytochrome and flowering time 1 (PFT1), PIF4, PIF5, and PIF7 act downstream of PHYB to promote flowering through the transcriptional activation of *FT* [25, 26]. These findings show that, in response to red light, PHYB integrates a complex network containing both flowering activators and repressors, with CO/FT as the likely ultimate targets of this light-induced signaling. Despite these insights, the mechanisms by which red light affects the flowering of DN rose plants have not been elucidated.

Sensitivity to red light reduced 1 (SRR1) is a pioneer protein whose sequence is very well conserved among a wide range of species, including mammals. The *AtSRR1* gene was identified as a regulator of AtPHYB signaling and is required for normal circadian clock function in *Arabidopsis*. The loss-of-function *Arabidopsis srr1* mutant displays an impaired circadian clock output and red-light response, as well as early flowering, especially under SD conditions, showing a reduced sensitivity to day-length. Consistent with this, the expression of several *FT* repressors, including *cycling DOF factor 1* (*CDF1*), *tempranillo 1* (*TEM1*), *TEM2*, and *flowering locus C* (*FLC*), is inhibited by *SRR1* silencing [27]. AtSRR1 was also found to control flowering time independently of photoperiod by functioning in the circadian clock and gibberellic acid pathways [28, 29]. In *Brassica rapa*, *BrFLC2* was identified as the gene underlying a key flowering quantitative trait locus related to *BrSRR1* [30]. *Brassica napus* has five *BnSRR1* genes with diverse functions, and transcriptional analysis identified BnCDF1 as the key target of BnSRR1 in flowering regulation [31]. Therefore, SRR1 proteins are considered integrators of the photoperiod and other pathways in the prevention of premature flowering when conditions were unsuitable for reproduction. However, whether RcSRR1 plays a role in the regulation of flowering and the response to red light in rose plants remains unknown.

The constitutive photomorphogenesis 9 (COP9) signalosome (CSN) is a relatively conserved protein complex in higher plants, which classically includes eight subunits (referred to as CSN1–CSN8). The CSN complex inhibits the activity of cullin (CUL)–RING E3 ubiquitin ligases (CRLs) by promoting the cleavage of Nedd8/Rub1 from CULs in a process known as deneddylation [32, 33]. CSN function might be therefore linked to protein degradation through the ubiquitin/26S proteasome system, through which the target proteins would be recognized and ubiquitinated by CRLs. *Arabidopsis csn* null mutants lack deneddylation activity and accumulate hyperneddylated CULs, resulting in a constitutive photomorphogenesis phenotype, establishing the CSN complex as an important component of light signaling [34, 35]. *Arabidopsis csn1* mutants display an abnormal floral transition with reduced AP3 transcription and a decrease in CUL1 deneddylation, suggesting that CSN plays an essential role in flower development. CSN was shown to interact with the Skp1-cullin-F-box protein (SCF) family of E3 ubiquitin ligase complexes [32, 36]. Further genetic evidence revealed that CSN is crucial for the function of the F-box protein unusual floral organs (UFO) in the promotion of APETALA3 (AP3) activity and floral identity, potentially via the CUL deneddylation of SCF^UFO^ [37]. In addition to the regulation of flowering, CSN has various functions in multiple biological processes, such as cell proliferation, gene regulation, self-incompatibility, abiotic and biotic stress responses, and light and hormone signaling [38–40]. To date, few CRLs have been identified as the direct targets of CSN in the regulation of flowering.

As transposase-derived transcription factors, far-red elongated hypocotyl 3 (FHY3) and its homolog far-red impaired response 1 (FAR1) play important roles in far-red light signaling mediated by PHYA. They negatively regulate flowering in *Arabidopsis* by activating the expression *Early Flowering 4* (*AtELF4*) under both SD and LD conditions [41–43]. FHY3 and FAR1 suppress the floral regulators *fruitful* (*FUL*), *leafy* (*LFY*), *AP1,* and *MIR172C*, through interacting with the squamosa-promoter binding protein-like 3 (SPL3), SPL4, and SPL5 transcription factors. They are therefore hypothesized to integrate environmental cues (light) with the developmental program (aging pathway) through the miR156–SPL module [44]. In addition to FAR1 and FHY3, another 12 FHY3/far-related sequence (FRS) genes have been identified in the *Arabidopsis* genome, which have been shown to play distinct roles in light signaling and the modulation of flowering time [45–47]. Genome-wide analyses have revealed many FRS-like genes in rose plants, with 91 in identified *Rosa wichuraiana* cv ‘Basye’s Thornless’ and 52 in *R. chinensis* cv ‘Old Blush’ [48]. The dispersed distribution and expression patterns of this expanded gene family might facilitate the regulation of shoot growth and flowering time in roses.

Understanding the effect of light spectral quality on rose growth and development is critical for selecting a supplementary lighting system for greenhouse cultivation. We previously demonstrated that the complementary expression of constans-like 4 (RcCOL4) under short-day (SD) and constans (RcCO) under long-day (LD) mainly contributed to DN response of rose (*Rosa chinensis*) plants [49]. In contrast to daylength, light intensity significantly affected the flowering time of rose, which was delayed at lower light irradiance (92 μmol m^−2^ s^−1^) compared with flowering at higher light irradiance (278 μmol m^−2^ s^−1^). Under low light intensity, phytochrome-interacting factor (PIF) proteins are stabilized and can form an RcPIFs-RcCO complex, thereby diminishing the availability of free RcCO required for the induction of *flowering locus T* (*RcFT*) and ultimately flowering [50]. For this reason, commercial growers commonly use supplementary light to improve both the quality and production of rose flowers, especially when not much natural light is available. In this study, we investigated the effects of different wavelengths of light produced by LEDs with the same photosynthetic photon flux density (PPFD) on rose flowering. We found that the red light (600–700 nm) inhibited rose flowering compared with blue (420–520 nm) or white (400–800 nm) light. A comprehensive analysis revealed that the RcFRSL3–RcSRR1–RcCSN5B module might regulate rose flowering under different light conditions by targeting the floral integrators CO and FT. These results reveal a novel mechanism underlying both rose flowering and the light response in plants.

## Results

### *RcSRR1* is required for the red light-mediated delay in flowering

**Figure 1:**
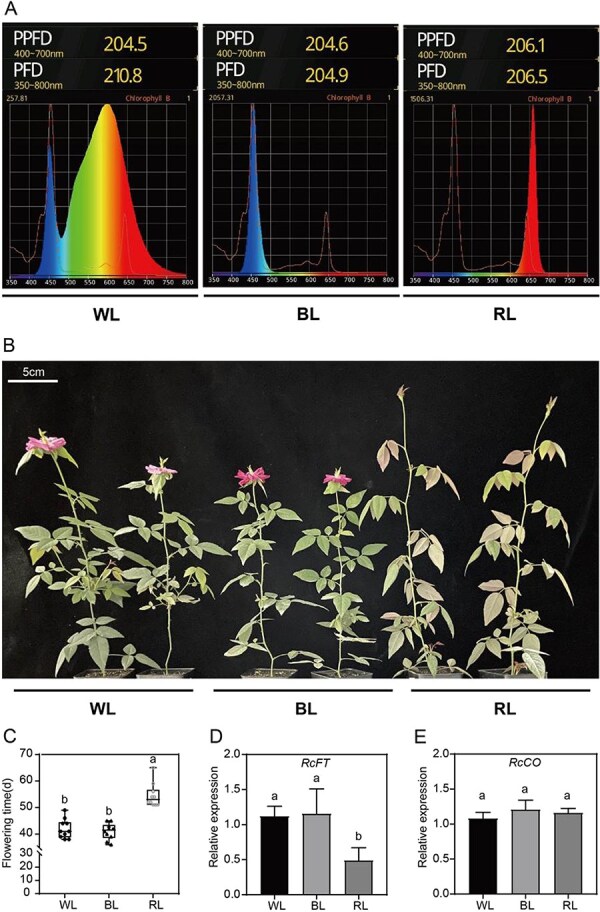
Red light delays flowering of *R. chinensis.* A. Spectral photon distributions and PPFD in WL, BL, and RL treatment. B. Flowering phenotypes and C. flowering time of rose cuttings under WL, BL, and RL. D. The relative expression levels of *RcFT* and E. *RcCO* in rose cuttings under WL, BL, and RL. The relative expression levels were determined by RT-qPCR with *RcGAPDH* as a reference gene. The representative result was shown from three-time repetitions. Mean value ± standard deviation was shown from at least 10 plants for flowering time, and from 3 biological replications each with three technical replications for gene expression. The different letters meant significant differences at *P* < 0.05 conducted with one-way ANOVA followed by Tukey’s multiple range test. WL: White light; BL: Blue light; RL: Red light

To begin to dissect the effects of different light conditions on rose flowering, we grew single-stemmed cuttings of *R. chinensis* cv ‘Old Blush’ in incubators equipped with white-light (400–800 nm), blue-light (420–520 nm) and red-light (600–700 nm) LEDs with the same PPFD (Fig. 1A). We then determined their flowering time and the relative expression levels of target genes. As shown in Fig. 1B and C, rose plants under red light treatment exhibited a prominent delayed flowering phenotype, with an average flowering time of 54.5 days, while the average flowering times of roses grown under white light and blue light were 42 and 41 days, respectively. Furthermore, the transcript level of *RcFT* but not *RcCO* was significantly lower in plants grown under red light than in those grown under either white or blue light (Fig. 1D and E).

To further find the potential regulators of red light-mediated rose flowering, we first examined the expression levels of several genes whose homologs have established roles both in flowering and red-light signaling in *Arabidopsis* [27, 51–54], and revealed that only the transcript level of *RcSRR1* is significantly higher under red light than under white or blue light (Supplemental Fig. S1), suggesting that *RcSRR1* may play a significant role in red light–mediated flowering in rose. A subsequent phylogenetic analysis using *SRR1* orthologs from other species (Supplemental Fig. S2) indicated that *RchiOBHm_Chr7g0215511* is most likely to be *RcSRR1* according to the *R. chinensis* ‘Old Blush’ Hm r2.0 genome database (https://lipm-browsers.toulouse.inra.fr/pub/RchiOBHm-V2/). We therefore cloned the coding sequence and conserved fragment of this gene and focused it for further study.

**Figure 2:**
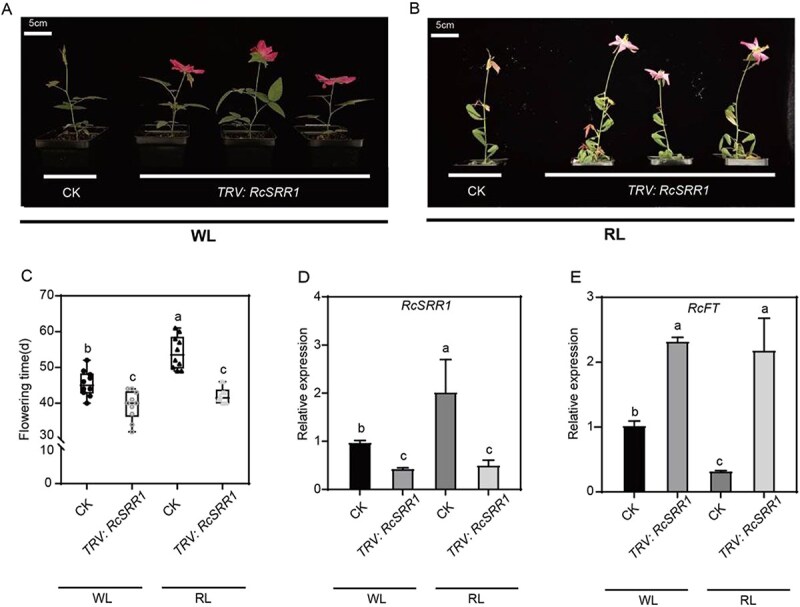
Silencing of *RcSRR1* leads to rose early flowering under both WL and RL. A and B. Flowering phenotypes of *RcSRR1*-silencing rose cuttings (*TRV*: *RcSRR1*) compared with control (CK) under WL and RL. The representative result was shown from three-time repetitions. C. Flowering time of rose cuttings. D. Relative expressions of *RcSRR1* and E. *RcFT* in CK and *TRV*: *RcSRR1* rose cuttings under WL and RL. The relative transcriptions were determined by RT-qPCR with *RcGAPDH* as a reference gene. Mean value ± standard deviation was shown from at least 10 plants for flowering time, and from three biological replications each with three technical replications for gene expression. The different letters meant significant differences at *P* < 0.05 conducted with one-way ANOVA followed by Tukey’s multiple range test. WL: White light; RL: Red light

*AtSRR1* is an integrator of flowering in *Arabidopsis* [29], but the biochemical properties of *RcSRR1* in rose have not previously been characterized. To elucidate the function of *RcSRR1*, we silenced *RcSRR1* in *R. chinensis* ‘Old Blush’ cuttings using VIGS (Fig. 2). RcSRR1-silenced cuttings showed an early flowering phenotype under both white and red light, although this response was much stronger under red light (Fig. 2A–C). The average flowering time of the *RcSRR1*-silenced cuttings was 39.4 d under white light, compared with 45.5 d in the wild type, while under red light the average flowering time of *RcSRR1*-silenced cuttings was 42.2 d, compared with 54.2 d in the wild type (Fig. 2C). We confirmed that *RcSRR1* expression was significantly decreased in the *RcSRR1*-silenced plants (Fig. 2D), which also showed higher *RcFT* expression than wild-type plants (Fig. 2E). By contrast, the transcript levels of the *FT* activator *RcCO* were not affected by *RcSRR1* silencing (Supplemental Fig. S3). Furthermore, the flowering time of *RcSRR1*-silenced rose cuttings did not differ between white- and red-light conditions (Fig. 2A–C), indicating that the flowering response to red light was totally abolished by *RcSRR1* silencing. These results indicate that RcSRR1 is required for the red light-mediated inhibition of flowering in rose plants, defining RcSRR1 as a flowering repressor but a positive regulator of red-light signaling.

### RcSRR1 interacts with RcCSN5B to facilitate RcCO degradation

As the CO−FT module is conserved in most plants, and integrates different flowering pathways [55], we next examined the possible protein interaction between RcSRR1 and RcCO as well as the possible activation of the *RcFT* promoter by RcSRR1. We showed that RcSRR1 does not directly interact with RcCO or *RcFT* (Supplemental Fig. S4), suggesting that it might affect rose flowering time by targeting components upstream of the RcCO−RcFT module.

**Figure 3:**
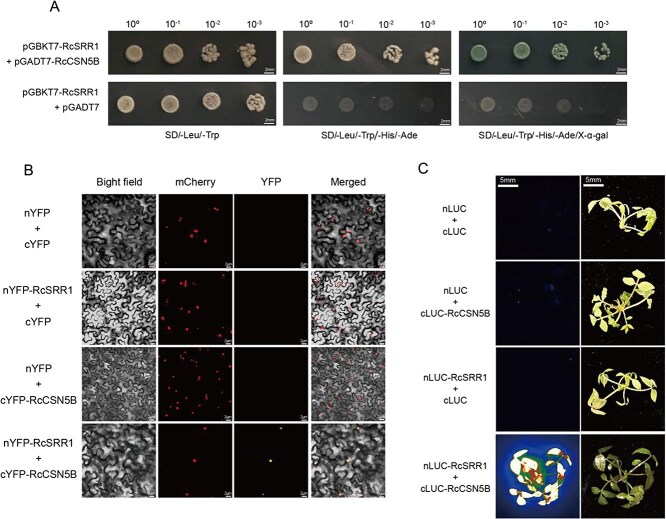
RcSRR1 physically interacts with RcCSN5B. A. Interaction assay between RcSRR1 and RcCSN5B by yeast two-hybrid. The coding sequences of *RcCSN5B* was inserted into *pGADT7* vector containing activating domain and the coding sequence of *RcSRR1* was inserted into *pGBKT7* vector containing DNA-binding domain, the empty *pGADT7* vector was used as negative control. The binding of pGBKT7-RcSRR1 to pGADT7-RcCSN5B was determined by yeast cell growth on synthetic dropout nutrient medium lacking Trp, Leu, His and Ade containing 20 μg/ml X-α-gal (SD/-Trp/-Leu/-His/-Ade + X-α-gal), while that growth on SD/-Leu/-Trp was used as positive control. B. Interaction of RcSRR1 and RcCSN5B in BiFC assay. The combination of nYFP-RcSRR1 + cYFP-RcCSN5B was infiltrated into leaves of genetically modified *N*. *benthamiana* carrying nucleus-localized red florescent protein (mcherry) and imaged under a confocal microscopy. The empty nYFP or cYFP co-infiltrated with corresponding construct were used as controls. YFP, yellow florescent protein. Scale bar corresponds to 20 μm. C. Interaction assay between RcSRR1 and RcCSN5B by split LUC complementation in rose seedlings. The combinations of nLUC + cLUC, nLUC + cLUC-RcCSN5B, nLUC-RcSRR1 + cLUC, nLUC-RcSRR1 + cLUC-RcCSN5B were co-infiltrated into rose seedlings and imaged by a CCD camera. All the representative results above were shown from three-time repetitions

To further identify the elements that interact with RcSRR1 to control flowering time, we conducted a Y2H assay using RcSRR1 as bait and captured 12 interaction partner proteins (Supplemental Table. S1). One of these putative interaction partners was the RcCSN5B subunit of the CSN, which is known to perform CUL deneddylation to inhibit the CRL activity required for ubiquitin−mediated proteolysis. A phylogenetic analysis confirmed that *RchiOBHm_Chr6g0304891* encoded RcCSN5B (Supplemental Fig. S5), and subsequent one-to-one Y2H, split-LUC complementation, and BiFC analyses confirmed the protein association between RcSRR1 and RcCSN5B in rose (Fig. 3A-C).

**Figure 4:**
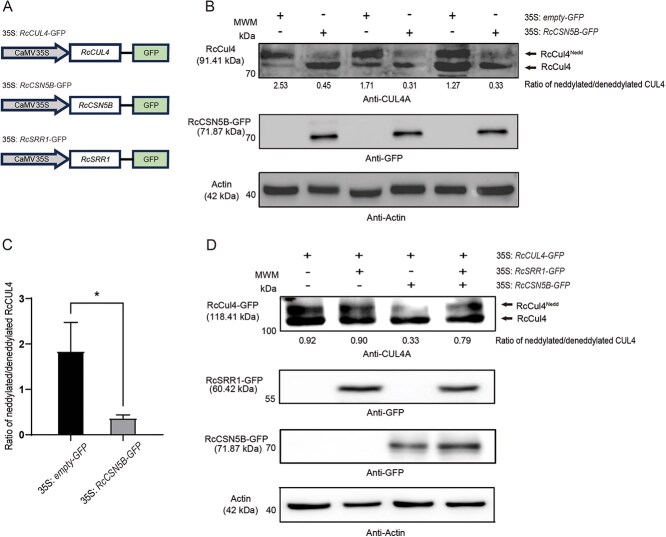
RcCSN5B shows deneddylation activity on RcCUL4. A. Schematic representation of *35S: RcCUL4*-*GFP*, *35S: RcCSN5B*-*GFP* and *35S: RcSRR1*-*GFP* constructs used for gene over-expression in rose seedlings. B. Neddylated and deneddylated RcCUL4 levels detected by Western Blotting in rose *calli* over-expressing *35S: empty-GFP* or *35S: RcCSN5B-GFP.* C. Ratios of neddylated/deneddylated RcCUL4 in rose *calli* shown in B. The asterisks represented statistically signification differences determined by Student’s *t-*test with *P < 0.05 as the threshold of significance. D. Neddylated and deneddylated RcCUL4 levels detected by Western Blotting in rose seedlings over-expressing *35S: RcCUL4*-*GFP* alone, or together with *35S: RcCSN5B-GFP* and *35S: RcSRR1-GFP* in different combinations. Band intensities in B and D were quantified by ImageJ ver 1.53c. The representative results were shown from three-time repetitions

As COP1 controls the CO protein stability [56], we next determined that RcCSN5B, RcCOP1, and RcCO interact with each other, likely forming a RcCSN5B−RcCOP1−RcCO complex (Supplemental Fig. S6). We thus next investigated the influence of RcSRR1 and/or RcCSN5B on the RcCOP1 E3 ubiquitin ligase activity through the RcCRL4−RcCOP1/RcSPA pathway. First, we cloned the coding sequence of *RchiOBHm_Chr5g0014271*, which was defined as *RcCUL4* based on a phylogenic analysis of this gene in *R. chinensis* and *A. thaliana* (Supplemental Fig. S7). Next, the coding sequences of *RcCUL4*, *RcSRR1*, and *RcCSN5B* without stop codons were inserted into a vector driven by the *CaMV 35S* promoter and followed by a GFP label at the 3′-end (Fig. 4A). These constructs were stably transformed in rose *calli* or transiently transformed into rose seedlings for the over-expressions of their corresponding genes. Western blotting with CUL4A−specific antibodies showed that the ratio of neddylated/deneddylated CUL4 was significantly lower in the *RcCSN5B* over-expression *calli* compared with control carrying empty vectors (Fig. 4B and C). Additionally, the ratio of neddylated/deneddylated CUL4 was 0.33 when *RcCSN5B* was over-expressed, compared with 0.92 in wild-type plants; and the neddylated/deneddylated ratio was almost unaffected by *RcSRR1* over-expression alone, but was 0.79 when *RcCSN5B* and *RcSRR1* were jointly over-expressed (Fig. 4D). These results indicate that RcCSN5B deneddylates RcCUL4, but this is negatively affected by RcSRR1.

CSN is known as a deneddylase of CRL E3 ligase activity. Its negative influence on the assembly of the CRL4^COP1^ E3 ligase complex has been shown to be conserved across species, from plants to mammals [32, 34–37, 57–59]. Our results suggest that RcSRR1 might promote the E3 ligase activity of the RcCRL4 − RcCOP1/RcSPA complex through its association with RcCSN5B, thereby enhancing RcCO proteolysis by RcCOP1/RcSPA and repressing *RcFT* activation and flowering.

**Figure 5:**
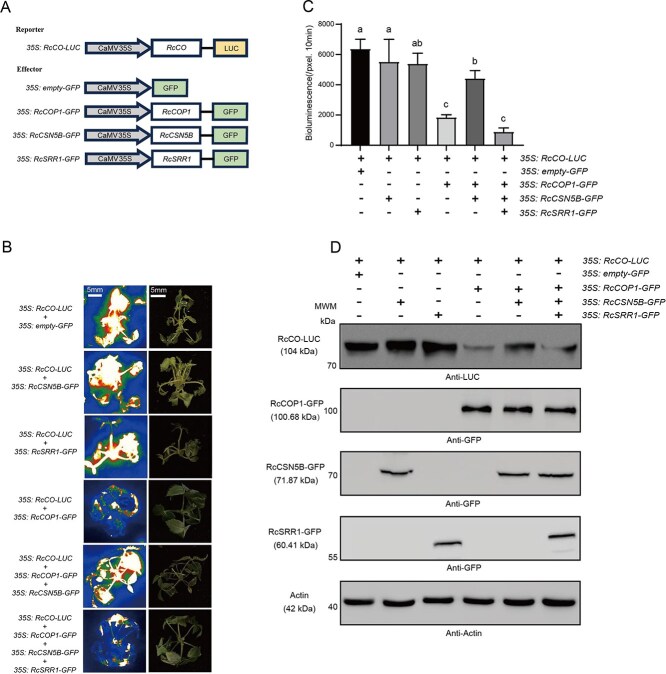
RcSRR1 and RcCSN5B antagonistically regulate RcCO protein stability. A. Schematic diagrams of *35S: RcCO*-*LUC*, *35S: empty*-*GFP*, *35S: RcCOP1*-*GFP*, *35S: RcCSN5B*-*GFP*, and *35S: RcSRR1*-*GFP* constructs for LUC assays. B. Representative images of LUC activities representing RcCO protein abundance in rose seedlings by the over-expression of *RcCO-LUC* with *empty-GFP*, *RcCOP1-GFP*, *RcCSN5B-GFP* or *RcSRR1-GFP* in different combinations. C. LUC intensities presented in B measured by Andor Solis ver 4.15. Mean value ± standard deviation was shown from three replications. The different letters meant significant differences at *P* < 0.05 conducted with one-way ANOVA followed by Tukey’s multiple range test. D. Western blotting results showing the RcCO-LUC fusion protein levels of the combinations described above in rose seedlings. Band intensities were quantified by ImageJ ver 1.53c. All the representative results in B and D were shown from three-time repetitions

To further elucidate this mechanism, we fused LUC to RcCO as a reporter and transiently co-expressed *35S: RcCO-LUC* with either an empty vector or with different combinations of *35S: RcSRR1-GFP*, *35S: RcCSN5B-GFP*, and *35S: COP1-GFP* as effectors (Fig. 5A) in rose cuttings [49]. Each construct has been verified to have been successfully transformed and to exhibit a consistently over-expression efficiency among its corresponding treatments, as shown in Supplemental Fig. S8. As shown in Fig. 5B and C, the RcCO protein level (LUC activity) was suppressed when *35S: RcCO-LUC* was co-expressed with *35S: RcCOP1-GFP* compared with its co-expression with the empty vector, demonstrating RcCOP1−mediated RcCO proteolysis. The addition of *35S: RcCSN5B-GFP* expression alongside *35S: RcCO-LUC* and *35S: COP1-GFP* expression led to significantly greater RcCO protein abundance, however, indicating that the RcCSN5B inhibits the activity of the RcCOP1 E3 ligase complex. The further addition of *35S: RcSRR1-GFP* alongside the co-expression of *35S: RcCO-LUC*, *35S: RcCOP1-GFP*, and *35S: RcCSN5B-GFP* reduced the RcCO protein level to some extent, confirming the adverse effect of RcSRR1 on RcCSN5B function. The similar RcCO protein levels in the plants co-expressing *35S: RcCO-LUC* with *35S: RcSRR1-GFP*, or *35S: RcCSN5B-GFP* eliminated the possibility of RcSRR1 and RcCSN5B directly affecting RcCO protein abundance. These results were in agreement with that from a Western Blotting analysis using LUC-specific antibodies (Fig. 5D). Taken together, these results further demonstrate that RcSRR1 negatively regulates RcCO protein stability by interfering with RcCSN5B *in vivo*.

### RcSRR1 suppresses *RcFT* transcription by disrupting RcCO stability

**Figure 6:**
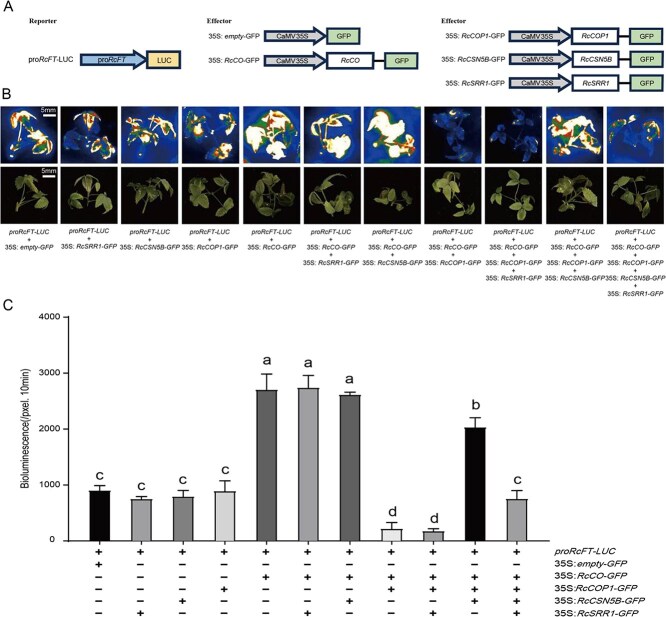
RcSRR1 suppresses *RcFT* transcription through interfering with RcCO stability. A. Schematic diagrams of *proRcFT-LUC*, *35S: empty-GFP*, *35S: RcCO-GFP*, *35S: RcCOP1-GFP*, *35S: RcCSN5B-GFP*, and *35S: RcSRR1-GFP* constructs for LUC assays. B. Representative images of *proFT-LUC* activity in rose seedlings infiltrated with empty vector or co-infiltrated with the constructs shown above in different combinations. The representative result was shown from three-time repetitions. C. The LUC intensity of each treatment shown in B. measured by Andor Solis ver 4.15. Mean value ± standard deviation was shown from three replications. The different letters meant significant differences at *P* < 0.05 conducted with one-way ANOVA followed by Tukey’s multiple range test

In the conserved CO–FT module, CO transcriptionally activates of *FT*. As RcSRR1 did not directly activate the *RcFT* promoter (Supplemental Fig. S4), we tested the effects of different protein combinations on *RcFT* expression. We fused LUC to the *RcFT* promoter as a reporter to generate the *proRcFT-LUC* construct and used a transient transformation system to co-express *proRcFT-LUC* with either an empty vector or various combinations of protein-producing constructs, whose expression efficiency was shown to be consistent across the corresponding treatments (the same as those in Fig. 5 and Supplemental Fig. S8) in rose seedlings. An analysis of the LUC activity (here representing *RcFT* transcription) revealed that *RcFT* was specifically activated by RcCO rather than the other proteins (Fig. 6, Supplemental Fig. S9). The combination of *35S: RcCO* and *35S: COP1* produced LUC activity inferior to that of the empty vector, indicating that *RcFT* activation by RcCO was abolished, further confirming the degradation of RcCO mediated by RcCOP1. The addition of *35S: RcCSN5B* and/or *35S: RcSRR1* altered *RcFT* expression, with LUC activity similar to *35S: RcSRR1*, enhanced by *35S: RcCSN5B*, but inhibited by the combination of *35S: RcCSN5B* and *35S: RcSRR1*, which was consistent with their roles in RcCO protein stabilization (Fig. 6). Collectively, these outcomes demonstrate that RcSRR1 associates with RcCSN5B to regulate its deneddylation activity of the RcCRL4–RcCOP1 complex, which maintains the optimal protein levels of RcCO required for *RcFT* transcription and flowering at the appropriate time.

### RcFRSL3 inhibits *RcSRR1* transcription and responds to red light

**Figure 7:**
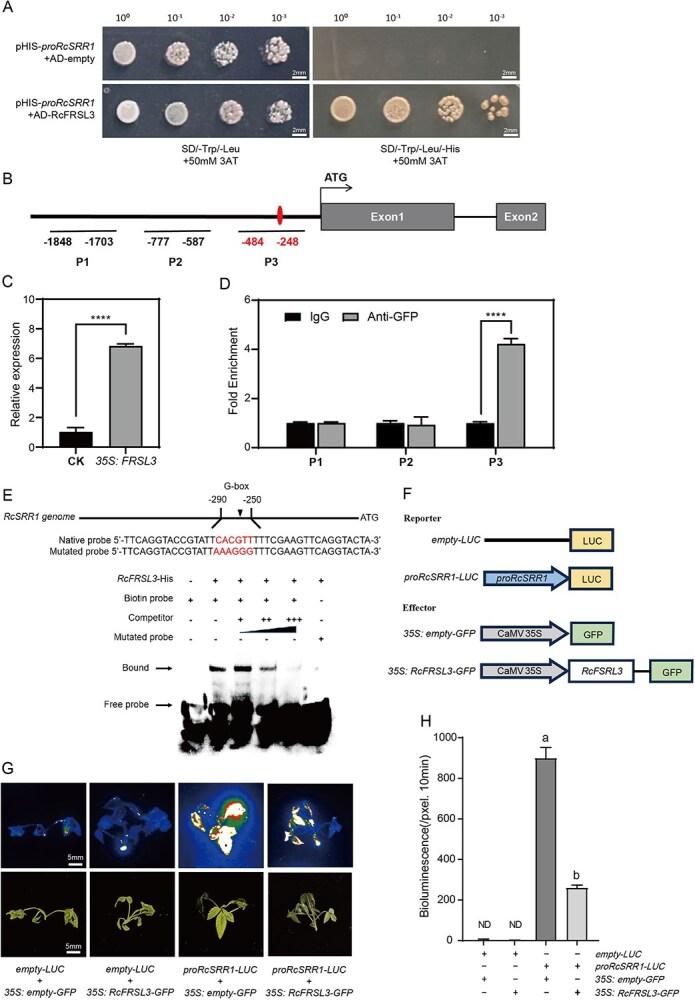
RcFRSL3 binds to *RcSRR1* promoter to inhibit its transcription. A. RcFRSL3 bound to *RcSRR1* promoter in a yeast-one-hybrid assay system. The binding of RcFRSL3-prey to *proRcSRR1*-bait was determined by yeast cell growth on synthetic dropout nutrient medium lacking Trp, Leu and His containing 50 mM 3-AT (SD/-Trp/-Leu/-His +50 mM 3-AT), while that growth on SD/-Trp/-Leu + 50 mM 3-AT was used as positive control. B. Schematic representation image showing ChIP-qPCR regions P1 (−1848 to −1703 bp), B (−777 to −587 bp) and C (−484 to −248 bp), marked by black bars below the *RcSRR1* genomic diagram. C. Relative expression level of *RcFRSL3* in *RcFRSL3* over-expressing rose seedlings. D. Relative enrichment levels of P1 to P3 fragments in *RcFRSL3* over-expressing rose seedlings by ChIP-PCR. IgG was used as a negative control. Mean value ± standard deviation was shown from 3 biological replications (*n* = 3) of transient transgenic rose seedlings. The asterisks represented statistically signification differences determined by Student’s *t-*test with ****P < 0.0001 as the threshold of significance. E. Binding assay of RcFRSL3 to the G-box motif within *RcSRR1* promoter in EMSA. F. Schematic diagrams of empty*-LUC*, *proRcSRR1-LUC*, *35S:* empty*-GFP*, and *35S: RcFRSL3-GFP* constructs used for LUC assays. G. Representative images of LUC activity in rose seedlings co-infiltrated *proRcSRR1-LUC* with *35S: empty-GFP* or *35S: RcFRSL3-GFP*. H. The LUC intensity of each treatment shown in G. measured by Andor Solis ver 4.15. Mean value ± standard deviation was shown from 3 replications (*n* = 3). The different letters meant significant differences at *P* < 0.05 conducted with one-way ANOVA followed by Tukey’s multiple range test. ND: Value not detected. The representative results above were presented from three-time repetitions

To identify the upstream transcription factors involved in the regulation of *RcSRR1* expression, we performed a Y1H assay using the *RcSRR1* promoter as bait. Of 22 candidates we identified (Supplemental Table S2), one gene (*RchiOBHm_Chr2g0118731*) was found to be a homolog of the *Arabidopsis* FRS-like family. This family is known to be involved in red light/far-red light signaling, so we subjected this candidate to further investigation. Based on a phylogenetic analysis of FRS-like genes from *R. chinensis* and *A. thaliana*, we named the candidate gene *far-red light–related sequence like 3* (*RcFRSL3*) (Supplemental Fig. S10). RcFRSL3 was predicted to be an FHY3/FAR1 family protein, containing a FAR1 DNA-binding domain (Supplemental Fig. S11). As AtSRR1 is a regulator of AtPHYB signaling pathway in *Arabidopsis* [27], we examined the relationship between RcFRSL3 and *RcSRR1* expression using a Y1H assay and found an interaction between RcFRSL3 and *RcSRR1* promoter (Fig. 7A). Afterwards, we tested the enrichment of three fragments containing light-regulating elements within the *RcSRR1* promoter by ChIP-PCR and found a greater enrichment in P3, which contains a G-box element (Fig. 7B-D, Supplemental Fig. S12). Then, an EMSA assay was carried out to confirm that RcFRSL3 specifically binds to the G-box within the *RcSRR1* promoter.(Fig. 7E). We next demonstrated that RcFRSL3 can suppress *RcSRR1* transcription (represented by LUC activity; Fig. 7F) by co-expressing *proRcSRR1-LUC* and *35S: RcFRSL3-GFP* (Fig. 7G and H). Consistent with this observation, RT-qPCR data also indicated that the transient over-expression of *RcFRSL3* in rose seedlings decreases *RcSRR1* expression (Supplemental Fig. S13), indicating that *RcFRSL3* is an upstream repressor of *RcSRR1*.

**Figure 8:**
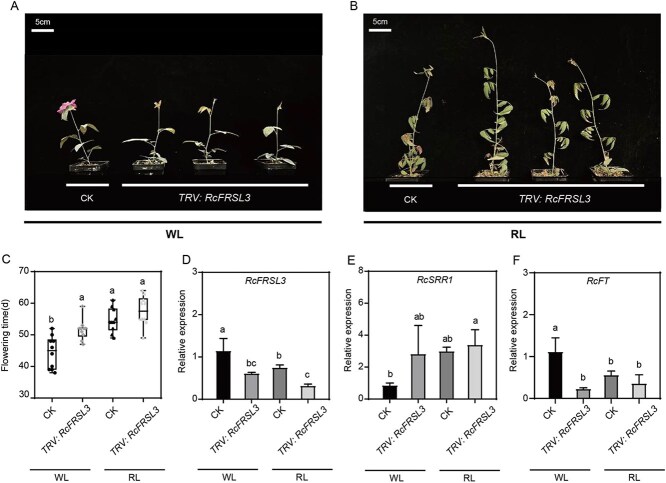
The silencing of RcFRSL3 delays rose flowering under both WL and RL. A and B Flowering phenotypes of *RcFRSL3*-silencing rose cuttings (*TRV*: *RcFRSL3*) compared with control (CK) under WL and RL. C. The flowering time and relative expressions of D *RcFRSL3*, E *RcSRR1*, and F *RcFT* in CK and *TRV*: *RcFRSL3* rose cuttings under WL and RL. The relative transcriptions were determined by RT-qPCR with *RcGAPDH* as a reference gene. The representative result was presented from three-time repetitions. Mean value ± standard deviation was shown from at least 10 plants for flowering time, and from three biological replications each with three technical replications for gene relative expression levels. The different letters meant significant differences at *P* < 0.05 conducted with one-way ANOVA followed by Tukey’s multiple range test. WL: White light; RL: Red light

Further silencing of *RcFRSL3* in rose cuttings delayed flowering and repressed the expression of *RcFT* under both white and red light, although the effect was less pronounced under red light (Fig. 8). RcFRSL3 could therefore be defined as a flowering activator but a negative regulator of the red light-mediated inhibition of flowering acting upstream of *RcSRR1*.

In summary, our findings provide a new understanding of flowering inhibition by red light signaling. Under white-light conditions, RcFRSL3 binds to the *RcSRR1* promoter to suppress its transcription and release its downstream targets to promote flowering. Upon red-light irradiations, the expression of *RcFRSL3* is reduced, leading to increased *RcSRR1* mRNA and protein levels. This, in turn, facilitates the interaction between RcSRR1 and RcCSN5B, promoting the RcCO proteolysis, and consequently inhibiting *RcFT* expression, thereby delaying flowering.

## Discussion

Plants receive a wide variety of external environmental cues and endogenous signals, which they must process in a cohesive manner to generate the optimal phenotype. Flowering is regulated in response to six main signals: temperature, photoperiod, vernalization, autonomous, gibberellin, and age [60]. The florigen gene *FT* and suppressor of overexpression of *CO1* (*SOC1*) represent the site of convergence for these flowering stimuli, and cause flower initiation by promoting the expression of the floral identity genes *LEAFY* (*LFY*) and *AP1* under the right conditions [60, 61]. Plants species can be divided into SD, LD, and DN groups based on their day length requirement for flowering, which is perceived by the photoperiod pathway [61, 62]. The CO–FT module appears to be conserved across all flowering plants so far, but its signaling output differs by species: AtCO transcriptionally activates *AtFT* under LD conditions in *Arabidopsis* (an inductive LD plant) [55], OsCO serves as a repressor of *OsFT* under LD in rice (*Oryza sativa*; an SD plant) [63], whereas RcCO and RcCOL4 alternately trigger *RcFT* expression under LD and SD condition in rose (a DN plant) [49]. CO is therefore the core protein coordinating light and circadian clock inputs to ensure that plants flower at the correct time, and its protein abundance must therefore be tightly controlled to optimize plant development. COP1, a RING finger E3 ubiquitin ligase, together with members of the SPA family (SPA1, SPA3, and SPA4), drives the ubiquitin-mediated proteolysis of CO at night, while nuclear COP1 is rapidly depleted upon light exposure to release CO [56]. This defines the temporal accumulation pattern of CO and confers the photoperiodic flowering response of plants.

The CSN subunits regulate various biological processes by deneddylating CRL to modulate its activities [38–40]. Although they are both repressors of photomorphogenesis, COP1 is not part of the CSN complex [64]. However, there is mounting evidence that their functions are strongly interconnected. CUL4-damaged DNA binding protein 1 (DDB1) interacts with DDB1 binding WD40 (DWD) protein to act as an E3 ligase, and CUL4–DDB1 physically associates with COP1–SPA complexes *in vitro* and *in vivo*, enhancing the formation of the CRL4^COP1-SPA^ E3 ubiquitin ligase complex despite COP1 independently possessing E3 ubiquitin ligase activity as well [58, 65, 66]. CUL4 also functions in collaboration with COP1/SPA to regulate photomorphogenesis and flowering [58]. Furthermore, the over accumulation of COP1 protein in the *cul4* and *cop9–1* mutants suggests the involvement of CUL4 and CSN in the degradation of COP1 [67]. In the present study, we determined that RcCSN5B, RcCOP1, and RcCO interact with each other, potentially forming an RcCSN5B–RcCOP1–RcCO complex (Supplemental Fig. S6). In our transient transformation experiment using rose cuttings, co-expression of *RcCOP1* and *RcCO* led to marked degradation of the RcCO protein, while the additional expression of RcCSN5B prevented RcCO degradation (Fig. 5). These results suggest that RcCSN5B is a positive regulator of RcCO stability, which might be attributed to the reduced RcCOP1 activity when considering the CRL4–COP1–SPA complexes.

Sun-light consists of different wavelengths of light with common or distinct roles in the photoperiodic regulation of flowering. Red light (high red:far-red ratios) delays flowering, while blue and far-red light (low red:far-red ratios) promote it [17]. In *Arabidopsis*, few downstream elements have hitherto been identified in the PHYB-mediated regulation of flowering. The transfer of vascular plant one-zinc finger protein to the nucleus from the cytoplasm may initiate the floral transition [68]. PFT1 downregulates CO transcription in response to red light [25], while PHL suppresses the inhibitory effect of PHYB on flowering time [24]. PHYB interacts with CO and the E3 ubiquitin ligase HOS1, thereby HOS1 promotes the red light–mediated degradation of CO in day time [23]. In *R. chinensis* cv ‘Old Blush’, we found that red light promotes *RcSRR1* expression and delays flowering (Fig. 1), while the silencing of *RcSRR1* hastens flowering under both red and white light, with a stronger response under red light (Fig. 2), suggesting that RcSRR1 acts as a flowering repressor but a red light–signaling activator. RcSRR1 physically interacts with RcCSN5B (Fig. 3), which functions in CRL deneddylation. To test the possible influence of RcSRR1 on RcCSN5B function, we transiently co-expressed *RcSRR1*, *RcCSN5B*, *RcCOP1*, and *RcCO* in rose cuttings, revealing that a reduction in RcCO protein level decreases *RcFT* transcription (Figs 5 and 6). This suggests that RcSRR1 represses the positive function of RcCSN5B in RcCO stabilization. Furthermore, the over-expression of *RcCUL4*, separately or together with *RcCSN5B* and *RcSRR1*, revealed that the RcCUL4 neddylation level is decreased by *RcCSN5B* but increased by the combination of *RcCSN5B* and *RcSRR1* (Fig. 4), demonstrating the negative role of RcSRR1 in RcCSN5B-mediated RcCUL4 deneddylation. Taken together, this genetic and biochemical evidence reveals that the red light–activated RcSRR1 interacts with RcCSN5B to reduce its deneddylation activity, thereby decreasing the RcCUL4 deneddylation of the RcCRL4^COP1-SPA^ complexes. This enhances the E3 ubiquitin ligase activity of COP1 and promotes RcCO proteolysis, with subsequent *FT* inactivation and delays in flowering. The present data reveal RcSRR1 to be an essential component in the red light (PHYB) signaling–mediated inhibition of rose flowering, which represents a previously uncharacterized molecular mechanism.

FHY3 and FAR1 were initially identified as essential components of PHYA-mediated far-red light signaling [41, 45]; however, they were also shown to play a critical roles in the responses to red light and UV-B light conditions [69, 70]. FHY3 is required for floral meristem determinacy and shoot apical meristem maintenance in the reproductive growth stage. An RNA sequencing analysis of FHY3-regulated genes revealed that many flower-specific genes are upregulated in the *fhy3* mutant, suggesting an important role for FHY3 in flower development [71]. Interestingly, the FRS-like gene family is considerably expanded in rose species, with 91 members in *Rosa wichuraiana* cv ‘Basye’s Thornless’ and 52 in *R. chinensis* cv ‘Old Blush’, whereas only 14 members have been detected in *Arabidopsis* [48]. This gene expansions in rose may have enabled the functional diversification of FRS-like genes within flowering regulation, with possible synergistic as well as antagonistic roles. In our Y1H screen for transcription factors that bind to *RcSRR1* promoter, we identified a FRS-like gene, *RcFRSL3*, which is downregulated by red light and specifically binds to the G-box within the promoter of *RcSRR1* to suppress its transcription (Fig. 7). The silencing of *RcFRSL3* by VIGS caused delayed flowering under both white- and red-light conditions, with less of an effect under red-light (Fig. 8). We therefore defined RcFRSL3 as a flowering activator but repressor of red-light signaling upstream of *RcSRR1*. These results demonstrate the important function of *FRS-*like genes in red-light signaling and rose flowering and will be of benefit in the elucidation of the evolutionary expansion of this gene family, especially in rose plants.

**Figure 9:**
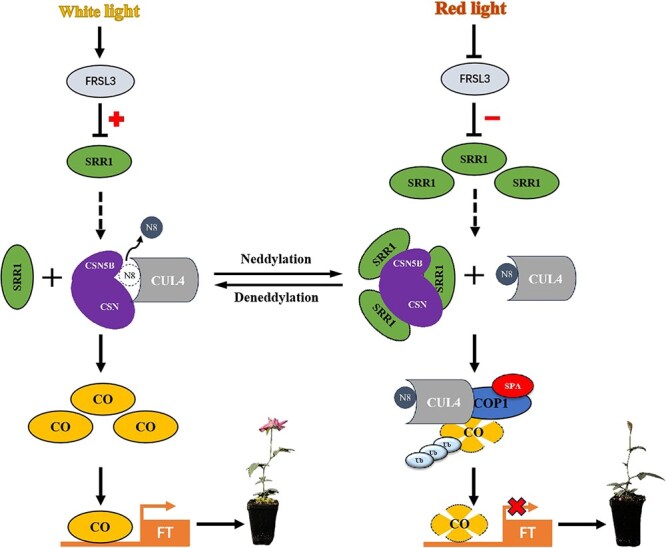
Simplified schematic model of rose flowering time regulated by RcSRR1. Under white-light conditions, RcFRSL3 binds to the *RcSRR1* promoter to suppress its transcription, and releases its downstream targets to promote flowering. Under red light conditions, the repression of RcFRSL3 to the transcription of *RcSRR1* is reduced with the reduction of RcFRSL3. The accumulated RcSRR1 protein associates with and interferes with RcCSN5B to decrease the RcCUL4 deneddylation, thereby enhances the E3 ubiquitin ligase activity of RcCRL4^COP1-SPA^ complex. This in turn promotes the RcCOP1/RcSPA-mediated RcCO proteolysis, which consequently diminishes the *RcFT* transcription and delays flowering of *R*. *chinensis*

In this study, we revealed the balance of flowering activators and repressors that safeguard appropriate flowering in rose under different light conditions (Fig. 9). Under white-light conditions, *RcSRR1* transcription is suppressed by the FRS-like family protein RcFRSL3 to liberate the downstream positive regulators of flowering. Rose plants perceive red-light signaling through the PHYB receptor to initiate downstream signaling, which first suppresses *RcFRSL3* expression and in turn releases RcSRR1 to associate with and inhibit RcCSN5B, a deneddylase that modulates CRL activity. The resulting decreased deneddylation level of RcCUL4 and increased E3 ubiquitin ligase activity of the RcCRL4^COP1-SPA^ complex enhances COP1/SPA-mediated RcCO proteolysis, and consequently diminishes the transcriptional activation of *RcFT* by RcCO, thus delays flowering. These results demonstrate that the RcFRSL3–RcSRR1–RcCSN5B module regulates flowering via ubiquitin-mediated RcCO proteolysis, offering a previously uncharacterized mechanism for the control of rose flowering by red-light signaling.

Collectively, the present results provide theoretical basis for the supplementary lighting in rose productive practice in greenhouse, as different light has distinct effect on rose flowering. However, the using of monochromatic light may have diverse effects on the growth and development of horticultural plants based on present reports, we therefore need to comprehensively assess the impacts on rose flowering of poly-chromatic light with different wavelengths and ratios in further study, and thus optimize an efficient illumination formula to promote rose production.

## Materials and methods

### Plant materials and growth conditions

*R. chinensis* cv ‘Old blush’ was used in this study. To cultivate rose seedlings, young stems of ‘Old blush’ plants grown in the open field were collected as starting materials in tissue culture on an initial medium (4.74 g L^−1^ Murashige and Skoog salts, 30 g L^−1^ sucrose and 7.5 g L^−1^ agar, pH 5.8). After one month, the tissue was transferred onto a proliferation medium (4.74 g L^−1^ Murashige and Skoog salts, 1.0 mg L^−1^ 6-benzyladenine, 0.1 mg L^−1^ 1-naphthaleneacetic acid, 30 g L^−1^ sucrose, and 7.5 g L^−1^ agar, pH 5.8) for subculture. The resultant seedlings were transplanted onto a rooting culture medium (4.74 g L^−1^ Murashige and Skoog salts, 0.05 mg L^−1^ 1-naphthaleneacetic acid, 30 g L^−1^ sucrose, and 7.5 g L^−1^ agar, pH 5.8) to obtain rooted seedlings.

Rooted rose seedlings were transplanted into square pots containing substrate comprising vermiculite, perlite and nutritive soil (1:1:1, *v*:*v*:*v*), then grown in a growth chamber with controlled conditions of 25°C, 40% relative humidity, and 200 μmol m^−2^ s^−1^ of light intensity under a LD cycle (16 h light-8 h dark). Then, the middle part of rose stem was used for generating rooted cuttings under the same conditions. For light quality treatments, rooted rose cuttings grown in square pots were placed into the incubator and subjected to the growth conditions listed above, but with either white-light (400–800 nm), monochromatic red-light (600–700 nm) or blue-light (420–520 nm). The incubator customized from KESHENG Company (KESHENG, Ningbo, China), which includes four separate growth chambers equipped with independent temperature and humidity regulators and the same amount of LED lamp beads. The light spectra of all treatments were measured using a handheld spectrometer PG200N (UPRtek, Zhunan Township, Taiwan, China).

### Construction of plasmids

To generate over-expression constructs, the coding sequences of *RcSRR1* (*RchiOBHm_Chr7g0215511*), *RcCSN5B* (*RchiOBHm_Chr6g0304891*), *RcCO* (*RchiOBHm_Chr2g0164091*), *RcCOP1* (*RchiOBHm_Chr5g0001791*), and *RcCUL4* (*RchiOBHm_Chr5g0014271*) were amplified from ‘Old Blush’ cDNA and cloned into the *pFAST-R05* vector (http://www.psb.ugent.be/) driven by *CaMV 35S* promoter and followed by a GFP tag. To generate VIGS constructs to silence *RcSRR1* or *RcFRSL3*, gene-specific fragments of *RcSRR1* (403 bp) or *RcFRSL3* (378 bp) were cloned into the *pTRV2* vector to generate the *TRV-RcSRR1* and *TRV-RcFRSL3* constructs.

For transient luciferase (LUC) activity assays, 2000 and 1976 bp promoter sequences upstream of the translational start sites of *RcSRR1* and *RcFT*, respectively, were inserted into the *pBGWL7* vector (http://www.psb.ugent.be/) to generate the *proSRR1-LUC* and *proFT-LUC* constructs. For the detection of RcCO protein degradation, the coding sequence of *RcCO* without the stop codon and driven by the *CaMV 35S* promoter was inserted into *pBGWL7* to generate the *35S: RcCO-LUC* construct. For the electrophoretic mobility shift assay (EMSA), the coding sequence of *RcFRSL3* without stop codon was inserted into *pGEX4T-1* alongside a GST tag. For the yeast one-hybrid (Y1H) assay, the full-length coding sequence of *RcFRSL3* was inserted into the *pGADT7* vector (prey; AD) to generate the *RcFRSL3*-AD vector, and 330 bp fragments upstream and downstream of the G-box motif in the *RcSRR1* promoter were cloned into the *pHIS2* vector to generate the *proRcSRR1-pHIS* constructs.

To analyze protein–protein interactions, the coding sequences of *RcSRR1*, *RcCSN5B*, *RcCO*, and *RcCOP1* without stop codons were integrated into the *pMK7WG-nL-2* (*nLUC*) or *pMK7WG-cL-2* (*cLUC*) vectors (http://www.psb.ugent.be/) for the split-LUC complementation assays, or were fused into the *pSPYCE* (*cYFP*) or *pSPYNE* (*nYFP*) vectors to generate constructs for the bimolecular fluorescence complementation (BiFC) assays. These genes were inserted into the *pGBKT7* or *pGADT7* to generate the constructs used in the yeast two-hybrid (Y2H) assay. All the above reactions were performed with the primers listed in Supplemental Table S3.

### VIGS

VIGS was conducted according to the procedures described by Tian *et al.* [72] *Agrobacterium tumefaciens* harboring *TRV-RcSRR1*/*TRV-RcFRSL3* constructs or empty *pTRV1*/*pTRV2* vectors were cultured in yeast extract broth medium (supplemented with 20 mM acetosyringone, 50 mg L^−1^ gentamicin, 50 mg L^−1^ kanamycin, and 30 mg L^−1^ rifampicin) for no less than 8 h at 28°C with constant shaking at 180 rpm. The *A. tumefaciens* cells were collected by centrifugation for 6 min at 5000 rpm, and the cell pellets were re-suspended with a pipet in infiltration buffer (10 mmol L^−1^ MgCl_2_, 200 mmol L^−1^ acetosyringone, 10 mmol L^−1^ MES, 0.01% (*v*/*v*) Silwet-L77, pH 5.6). The suspension of *A. tumefaciens* cells carrying *pTRV1* was mixed with that harboring *TRV2-RcSRR1*, *TRV2-RcFRSL3* or empty *TRV2* in a 1:1 (*v:v*) ratio. Whole rose cuttings were then fully submerged into the mixtures and exposed twice to a − 25 kPa vacuum for 60 s each time. The infiltrated rose cuttings were transplanted into the substrates described above for flowering time inspection and gene expression analyses.

Flowering time was measured by recording the number of days from transplantation of rose cuttings to fully opening of petals. Each VIGS experiment was repeated for three times with similar results and at least 10 individuals were measured each time.

### Western blotting assay

For the protein abundance or neddylation assays, total proteins were extracted from rose seedlings or *calli* over-expressing the genes of interest using a plant protein extraction reagent kit (ComWin Biotech, Beijing, China). The extracted proteins were mixed with 2 × SDS sample buffer in a 1:1 ratio (*v*:*v*) and denatured by heating in a boiling water bath for 10 min before being used for western blotting. The proteins were then isolated in a 12.5% (w/v) polyacrylamide gel by polyacrylamide gel electrophoresis. Next, the protein samples in gel were electrically transferred onto a nitrocellulose membrane, and incubated for 6–8 h with gene-specific antibodies or label antibodies as primary antibodies, then with secondary antibodies for 2–4 h before being detected by a ChemiDoc MP imaging system (BIO-RAD Laboratories, Hercules, California, USA).

For neddylation assays, the specific antibody of CUL4A (Sigma-C0371) was ordered from Sigma-Aldrich Trading Co. Ltd (Shanghai, China), its specificity was verified by the western blotting, and the definition of neddylated or de-neddylated bands was conducted according the literature [57].

### Yeast hybrid experiments

To perform Y1H assays, the *proSRR1-pHIS* construct was co-transformed with the rose leaf cDNA library*-AD* constructs, *RcFRSL3-AD* construct or empty AD vector into yeast (*Saccharomyces cerevisiae*) strain Y187 cells using a yeast hybrid kit (Takara Bio). The transformed Y187 cells were grown on SD medium lacking tryptophan and histidine (SD/-Trp/-His) with filtered 3-amino-1,2,4-triazole (3-AT) as positive control, and grown on SD medium lacking tryptophan, leucine, and histidine (SD/-Trp/-Leu/-His) with 3-AT for screening of potential targets and examination of activation.

For the Y2H assays, combinations of either *RcCSN5B-AD*, or *RcCO-AD*, and *RcSRR1-BD*, *RcCOP1-BD*, or *RcCO-BD* (or the corresponding empty vector) were co-transformed into yeast strain Y2H Gold cells using a Y2H assay kit (Takara Bio). The transformed Y2H Gold cells were cultured on SD medium lacking histidine, leucine, and tryptophan (SD/-His/-Leu/-Trp) to screen potential targets, while that cultured on SD medium lacking leucine and tryptophan (SD/-Leu/-Trp) was used as the positive control. They were also cultured on SD medium lacking adenine, histidine, leucine, and tryptophan (SD/-Ade/-His/-Leu/-Trp) to examine the potential interactions.

### Plant transformation

Transient transformations were performed in rose seedlings of *R. chinensis* cv 'Old Blush' based on previous studies [73]. The apexes of rose seedlings cultured on proliferation medium were cut off and dipped into the mixed resuspension of *A. tumefaciens* strain GV3101 carrying different vectors. *A. tumefaciens* resuspensions were infected into rose seedlings by vacuum infiltration at 0.5 MPa for 5 min, and then kept on MS solid medium for 2–4 d before LUC activity assays.

To get transgenic rose *calli*, *calli* generated from *R. chinensis* cv 'Old Blush' seedlings was dipped into the mixed resuspension of *A. tumefaciens* strain GV3101 carrying different vectors and kept shaking at 100 rpm, 28°C for 40 min. Then, the infected rose *calli* was transferred to proliferation medium containing 400 mg/L TMT and 60 mg/L kanamycin for screening.

### Chromatin immunoprecipitation PCR

Chromatin immunoprecipitation PCR (ChIP-PCR) was carried out as described previously [74]. Rose seedlings over-expressing *RcFRSL3* were collected to obtain chromatin suspension, which was then sonicated into approximately 500 bp fragments. The lysate was precleared by incubation together with 50 μl of protein-A agarose beads/salmon sperm DNA (Millipore, Billerica, USA) for 1 h. After incubating with IgG and anti-GFP antibody (Abcam, Cambridge, UK) overnight, the bound DNA fragments were eluted and purified using the CTAB method and then quantified by qRT-PCR with the primers listed in Supplemental Table S3.

### Prokaryotic expression and purification of recombinant proteins

The constructs carrying target genes were transformed into *E. coli* strain BL21, and the positive clones were picked for protein induction. Then, the transformed BL21 was inoculated into 3 ml of LB medium containing corresponding antibiotic of the constructs and shaken overnight at 37°C with the speed set at 230 rpm in a shaking incubator. Afterwards, 1 ml of the suspension was transferred into 50 ml of LB medium with resistance, and continued to incubate in a shaking incubator at 37°C until OD600 = 0.5–0.8. Then, the suspension was supplemented with 100 mM/L IPTG for the target protein induction, and incubated overnight at 16°C with a shaking speed of 200 rpm. The bacterial pellet was collected by centrifugation and washed twice with 1 × PBS. PMSF was added to prevent protein degradation during the process. The target protein was obtained by disruption with a sonic dismembrator (Fisher Scientific 120, Thermo Fisher Scientific, USA) and purified using a protein purification kit (CWBIO, China).

### EMSA

For the EMSA, the G-box probe and its muted probe were synthesized with 3′-end biotin labeling, and unlabeled G-box probe was used as a competitor. About, 2 μg purified recombinant His-RcFRSL3 protein was co-incubated with the probe and muted probe and the competitor using a Light Shift Chemiluminescent EMSA kit (Thermo Fisher Scientific, Waltham, Massachusetts, USA) at 24°C for 20 min, according to the manufacturer’s instructions. The reaction products were subjected to electrophoresis on 6% (*w*/*v*) native PAGE gels in 0.5× TBE buffer. All the probe sequences used are listed in Supplemental Table S3.

### Statistical analyses

Statistical significances were determined using Student’s *t*-test for two-group data or one-way ANOVAs with Tukey’s multiple range test for multiple-group data. All means and error bars presented are means ± SEM of three or more experimental replications.

## Supplementary Material

Web_Material_uhaf025

## Data Availability

The data supporting the findings of this study are available within the paper and its online supplementary data.
