# Supplementary material for: RcSRR1 interferes with the RcCSN5B-mediated deneddylation of RcCRL4 to modulate RcCO proteolysis and prevent rose flowering under red light
Source: Hortic Res. 2025 Jan 21;12(5):uhaf025. doi: 10.1093/hr/uhaf025 (PMC11979331; doi:10.1093/hr/uhaf025)
Supplement: Web_Material_uhaf025 [file web_material_uhaf025.docx]

**Supplementary Figures S1-S11 and Table S1-S3**

**RcSRR1 interferes with the RcCSN5B-mediated deneddylation of RcCRL4 to modulate RcCO** **proteolysis and prevent rose flowering under red light**

**Running title:** RcSRR1 inhibits rose flowering under red light

Weinan Wang ^a, b, c, d^, Jingjing Sun ^a, b, c^, Chunguo Fan ^a, b, c^, Guozhen Yuan ^a, b, c^, Rui Zhou ^a, b, c^, Jun Lu ^a, b, c^, Jinyi Liu ^a, b, c^, Changquan Wang ^a, b, c*^

^a^ College of Horticulture, Nanjing Agricultural University, Nanjing 210095, China

^b^ Key Laboratory of Landscaping, Ministry of Agriculture and Rural Affairs, Nanjing 210095, China;

^c^ Key Laboratory of State Forestry and Grassland Administration on Biology of Ornamental Plants in East China, Nanjing 210095, China

^d.^ School of Civil Engineering, Yantai University, Yantai 264005, China

* Correspondence: Changquan Wang (cqwang@njau.edu.cn)


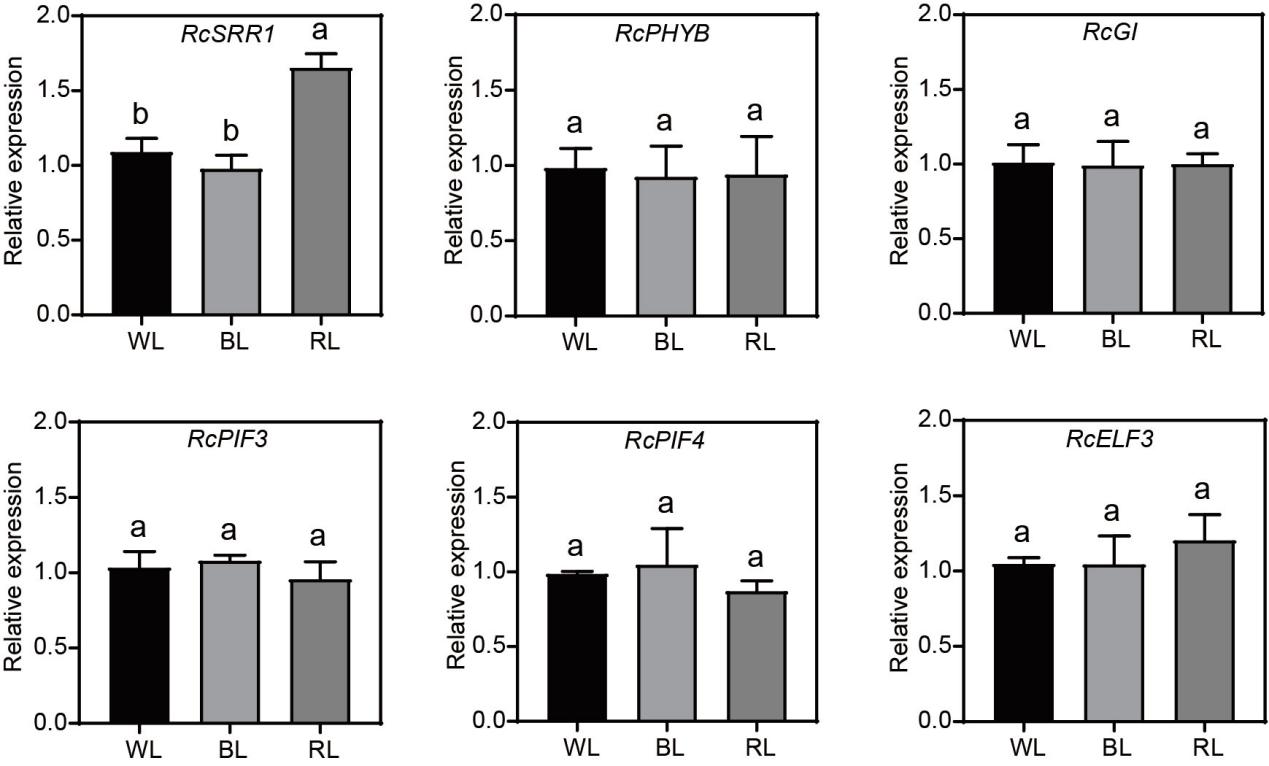


**Supplemental Figure S1 Relative expressions of selected genes with potential roles in red light-mediated flowering in *R. chinensis***.

These genes were selected based on their homologs with established roles both in flowering and red-light signaling in *Arabidopsis* according to previous studies (Huq et al., 2000; Gyula et al., 2003; Staiger et al., 2003; Pham et al., 2018; Ronald et al., 2022). All the relative expression levels were determined by RT-qPCR with *RcGAPDH* as a reference gene. Mean value ± standard deviation was shown from 3 biological replications (n=3) each with 3 technical replications. The different letters meant significant differences at *P*<0.05 conducted with one-way ANOVA followed by Tukey’s multiple range test. WL: White light; BL: Blue light; RL: Red light.


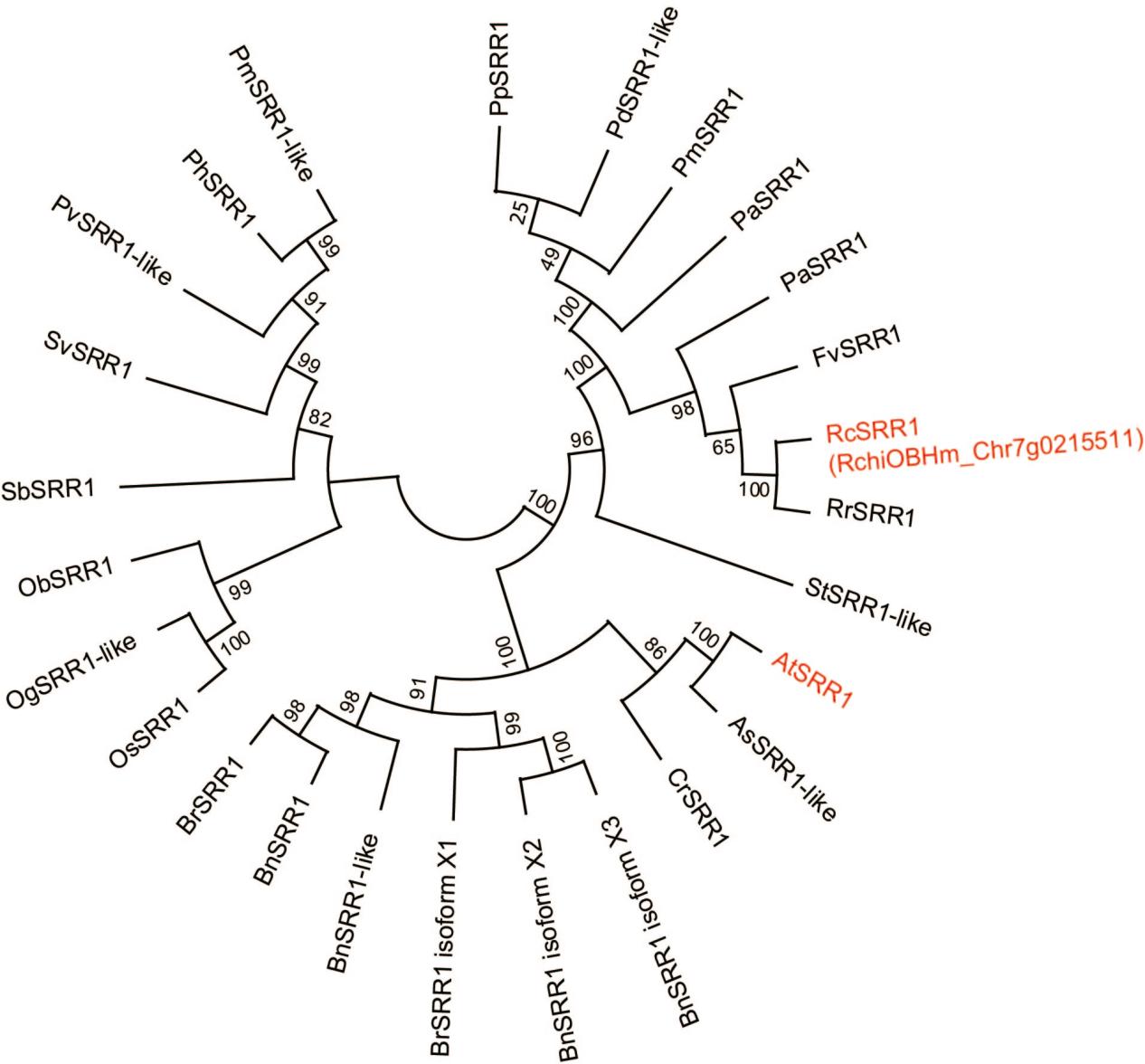


**Supplemental Figure S2 Phylogenetic tree of RcSRR1 and SRR proteins in other species.**

The phylogenetic analysis was performed based on the neighbor-joining method. Both the multiple sequences alignment and the phylogenetic tree construction were performed with MEGA version 11.0.13. The values at the nodes represented bootstrap values from 1000 replicates.


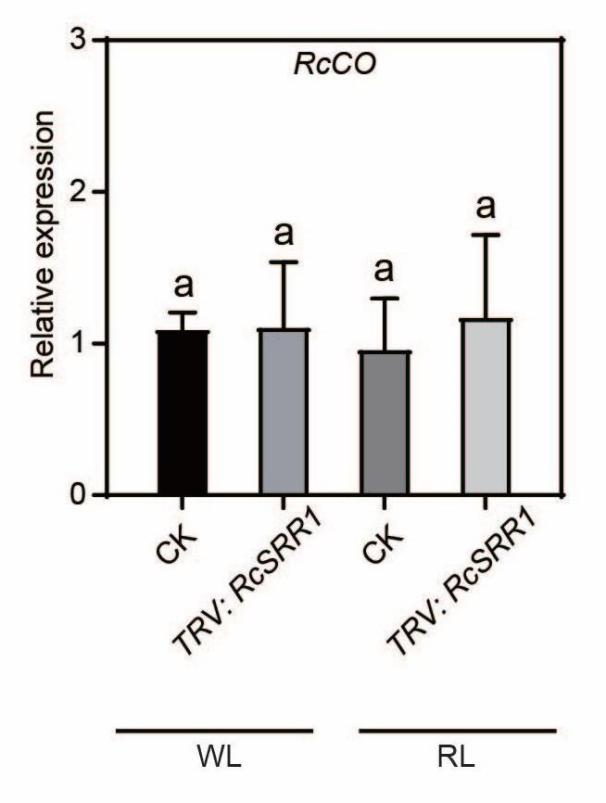


**Supplemental Figure S3 Relative expressions of *RcCO* in *RcSRR1*-silenced (*TRV: RcSRR1*) and control (CK) rose cuttings under WL and RL.**

All the relative expression levels were detected by RT-qPCR with *RcGAPDH* as a reference gene. Mean value ± standard deviation was shown from 3 biological replications (n=3) each with 3 technical replications. The different letters meant significant differences at *P*<0.05 conducted with one-way ANOVA followed by Tukey’s multiple range test. WL: White light; RL: Red light.


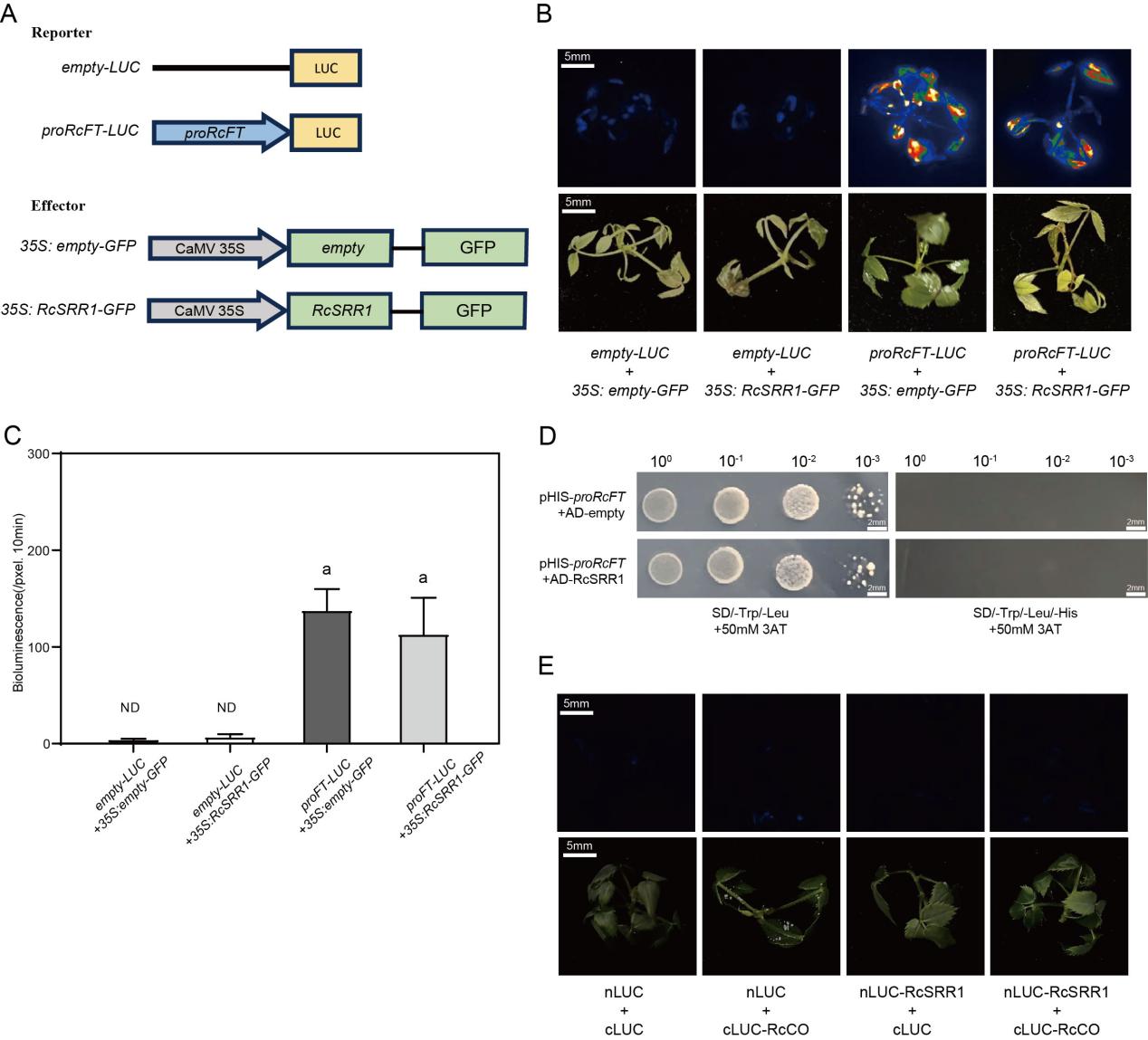


**Supplemental Figure S4 RcSRR1 did not bind to *RcFT* promoter or physically interact with RcCO**.

(A) Schematic diagrams showing the constructs used in LUC assays. *empty-LUC* and *proRcFT-LUC* were used as reporters, *35S: empty-GFP* and *35S: RcSRR1-GFP* were used as effectors. (B) Representative images of LUC assays showed *proRcFT-LUC* activity in rose seedlings. (C) The LUC intensity of each combination shown in (B) measured by Andor Solis ver 4.15. Mean value ± standard deviation was shown from 3 biological replications (n=3) of transient transgenic rose seedlings. Different letters above the columns denote statistically significant differences at *P*<0.05 determined by one-way ANOVA followed by Tukey’s multiple range test. ND: value not detected. (D) Yeast-one-hybrid assay system showing RcSRR1 did not bind to *RcFT* promoter. The binding of RcSRR1-prey to *proRcFT*-bait was determined by yeast cell growth on synthetic dropout nutrient medium lacking Trp, Leu and His containing 50 mM 3-AT (SD/-Trp/-Leu/-His), while that growth on SD/-Trp/-Leu + 50 mM 3-AT was used as positive control. (E) Interaction assay between RcSRR1 and RcCO by split LUC complementation in rose seedlings. All the representative results in **B.**, **D.** and **E.** was shown from three times of repetitions.


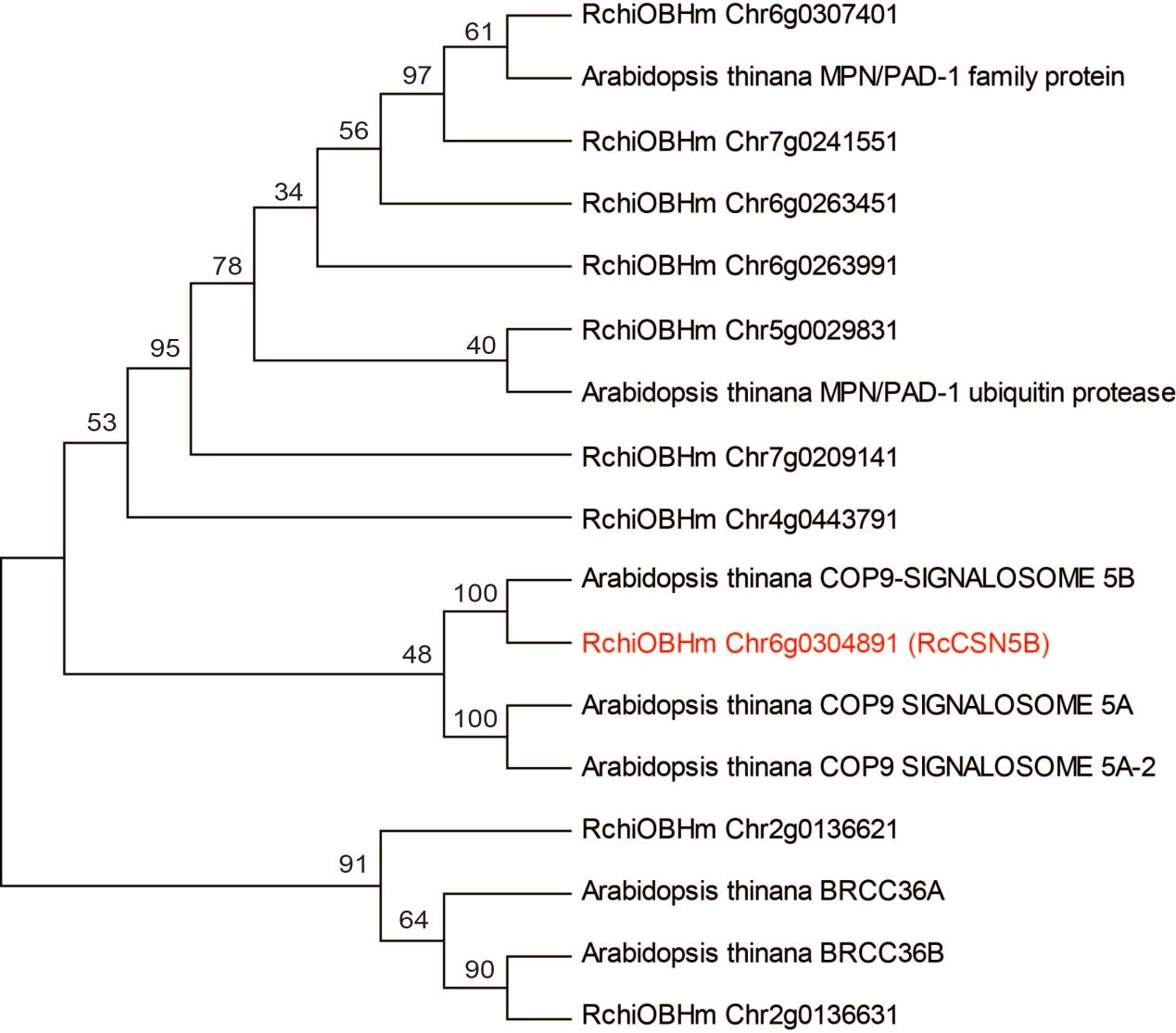


**Supplemental Figure S5 Phylogenetic tree of RcCSN5B and its homologous proteins in *R*. *chinensis* and *A*. *thaliana*.**

The phylogenetic analysis was performed based on the neighbor-joining method. Both the multiple sequences alignment and the phylogenetic tree construction were performed with MEGA version 11.0.13. The values at the nodes represented bootstrap values from 1000 replicates.


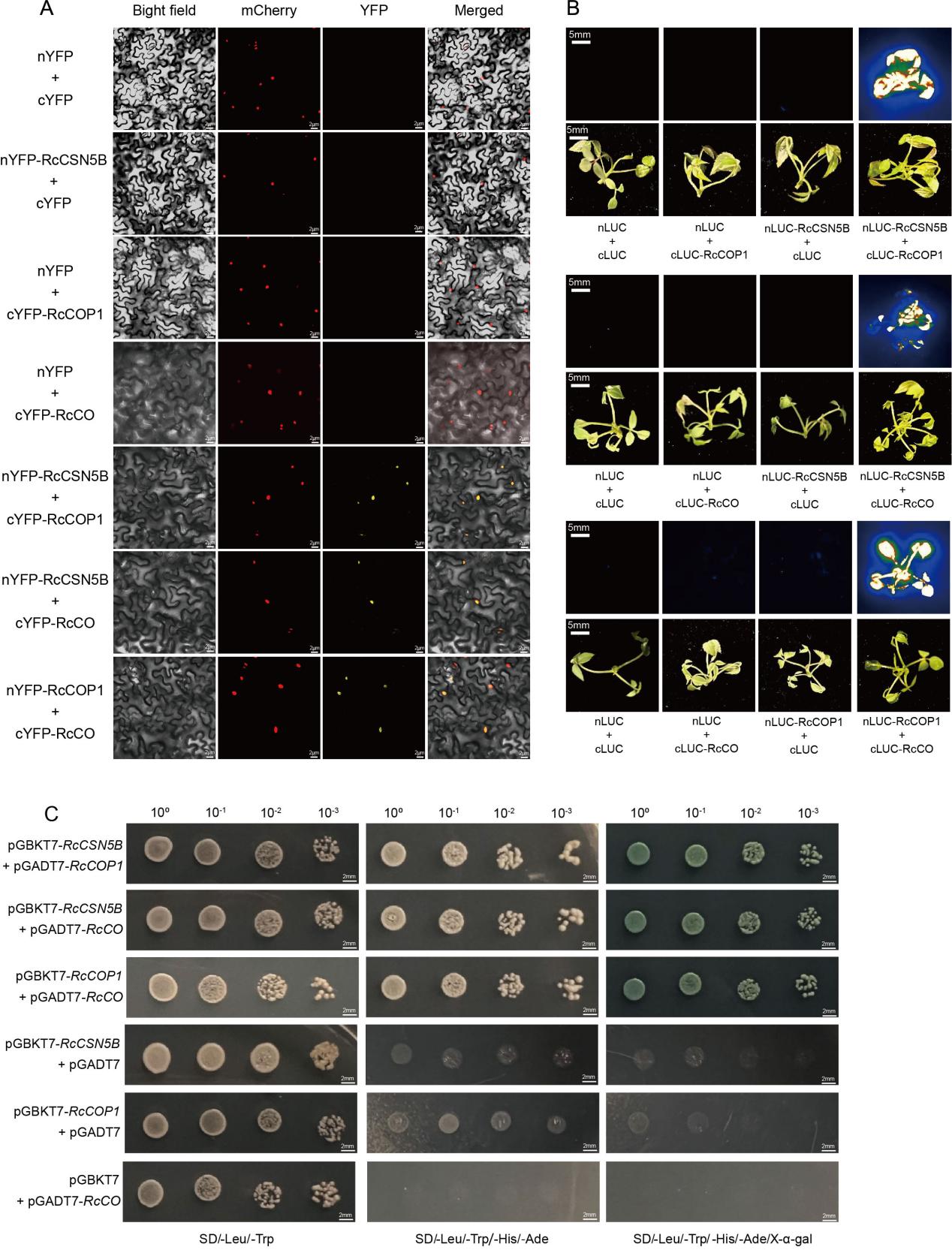


**Supplemental Figure S6 RcCSN5B, RcCOP1 and RcCO interacted with each other.**

(A) Interactions of RcCSN5B, RcCOP1 and RcCO in BiFC assays. The combinations of *nYFP* + *cYFP*, *nYFP-RcCSN5B*+*cYFP*, *nYFP* + *cYFP-RcCOP1*, *nYFP* + *cYFP-RcCO*, *nYFP-RcCSN5B* + *cYFP-RcCOP1*, *nYFP-RcCSN5B* + *cYFP-RcCO*, *nYFP-RcCOP1* + *cYFP-RcCO* were infiltrated into leaves of genetically modified *N*. *benthamiana* carrying nucleus-localized red florescent protein (mcherry). YFP, yellow florescent protein. Scale bar corresponds to 20 μm. (B) Interaction assays of RcCSN5B, RcCOP1 and RcCO by split LUC complementation in rose seedlings. The combinations of *nLUC-RcCSN5B* + *cLUC-RcCOP1*, *nLUC-RcCSN5B* + *cLUC-RcCO*, *nLUC-RcCOP1* + *cLUC-RcCO* were infiltrated into rose seedlings and imaged using a CCD camera. The empty *nLUC* or *cLUC* with either construct was used as control. (C) Interactions of RcCSN5B, RcCOP1 and RcCO in yeast two-hybrid assays. The coding sequences of *RcCSN5B* and *RcCOP1* were inserted into the *pGBKT7* vectors and the coding sequences of *RcCOP1* and *RcCO* were inserted into the *pGADT7* vectors, the empty *pGADT7* and *pGBKT7* vectors were used as negative controls. The bindings of pGBKT7-RcCSN5B to pGADT7-RcCOP1, pGBKT7-RcCSN5B to pGADT7-RcCO, pGBKT7-RcCOP1 to pGADT7-RcCO were determined by yeast cell growth on synthetic dropout nutrient medium lacking Trp, Leu, His and Ade containing 20 μg/mL X-α-gal (SD/-Trp/-Leu/-His/-Ade + X-α-gal), while that growth on SD/-Leu/-Trp were used as positive controls. All the experiments mentioned above were repeated 3 times with similar results, and the representative result was shown.


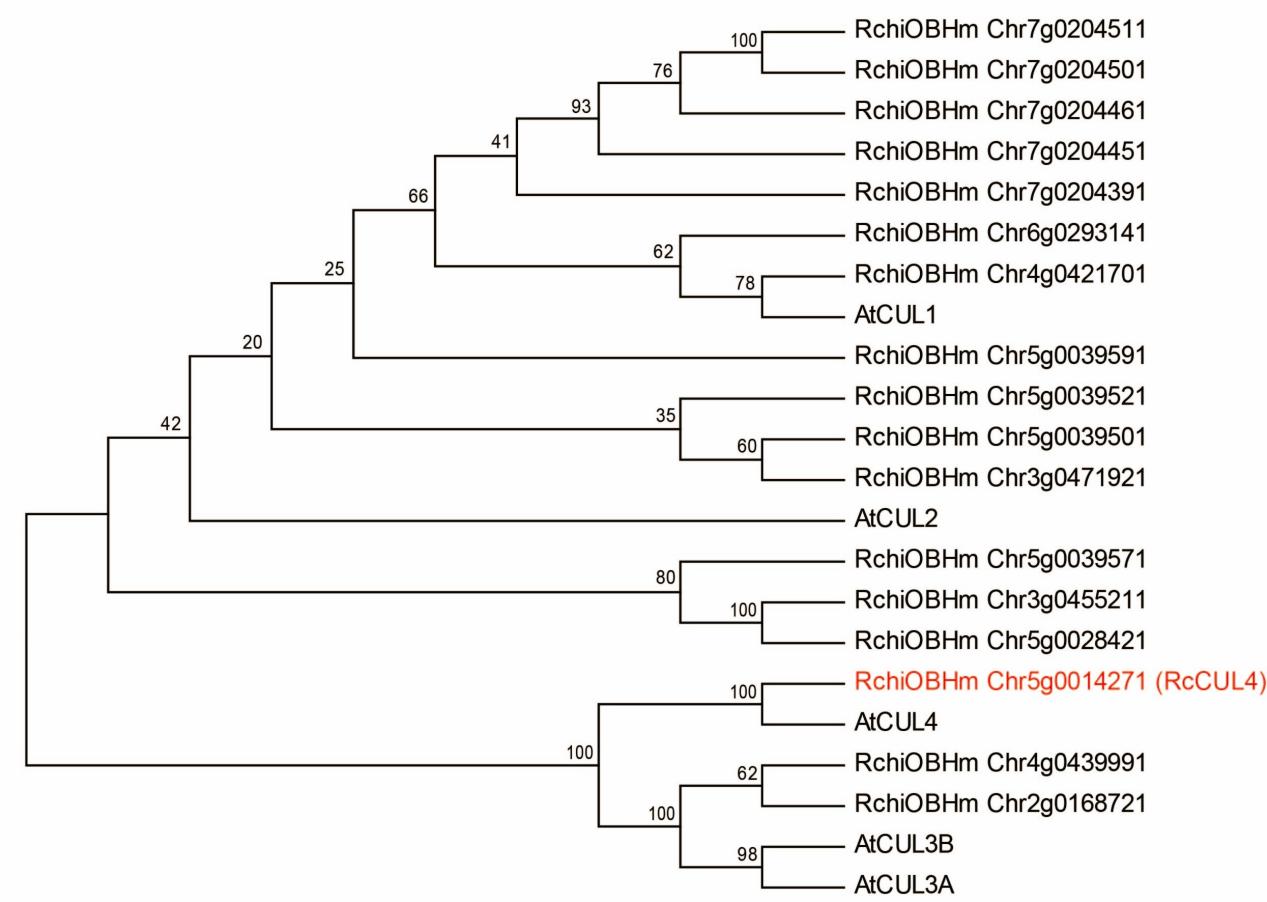
**Supplemental Figure S7 Phylogenetic tree of RcCUL4A and its homologous proteins in *R*. *chinensis* and *A*. *thaliana.***

The phylogenetic analysis was performed based on the neighbor-joining method. Both the multiple sequences alignment and the phylogenetic tree construction were performed in MEGA version 11.0.13. The values at the nodes represented bootstrap values from 1000 replicates.

**
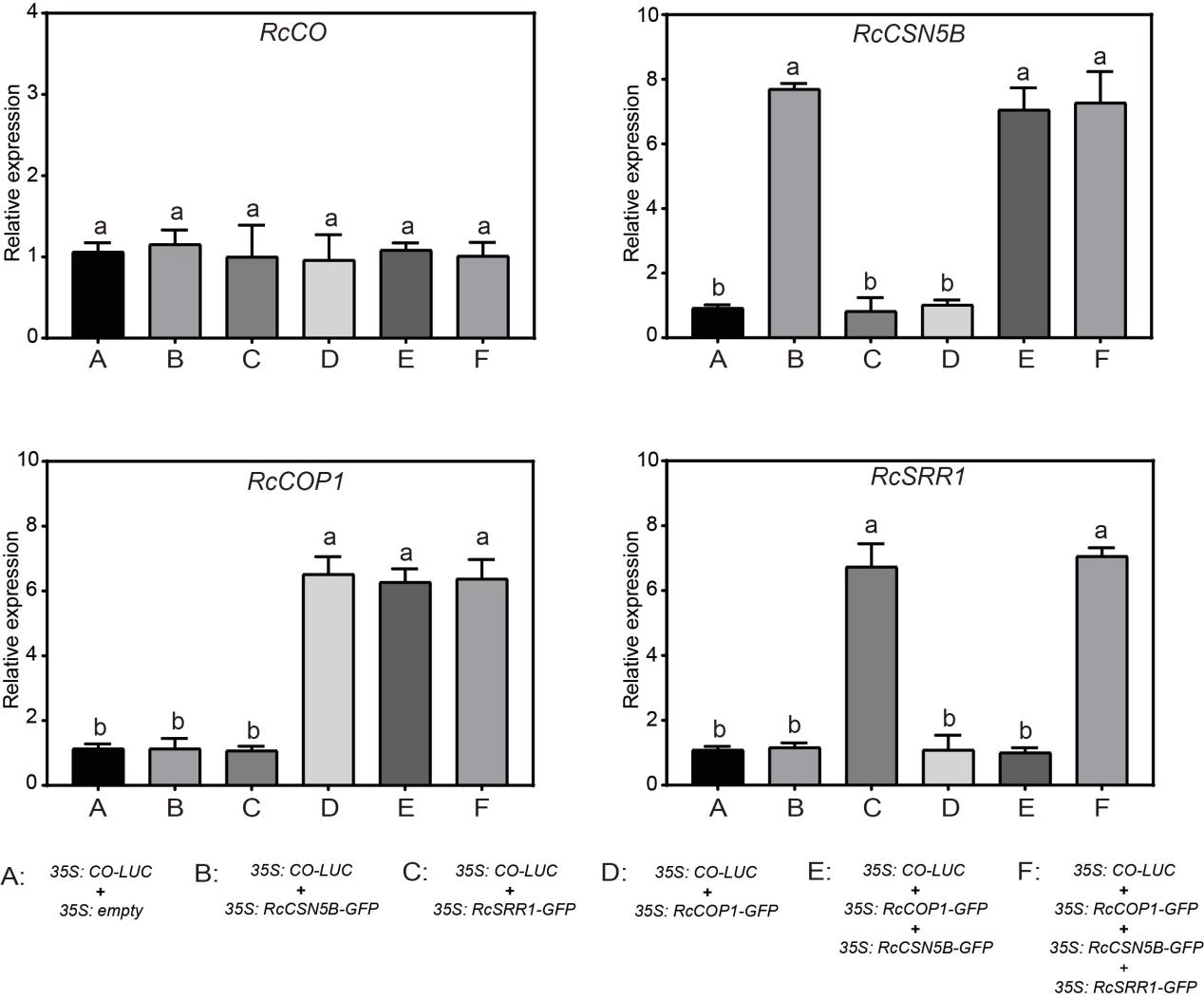
Supplemental Figure S8 Expression efficiency of individual construct in each treatment of Fig 5B**.

All the relative expression levels were determined by RT-qPCR with *RcGAPDH* as a reference gene. Mean value ± standard deviation was shown from 3 biological replications (n=3) each with 3 technical replications. The different letters meant significant differences at *P*<0.05 conducted with one-way ANOVA followed by Tukey’s multiple range test.


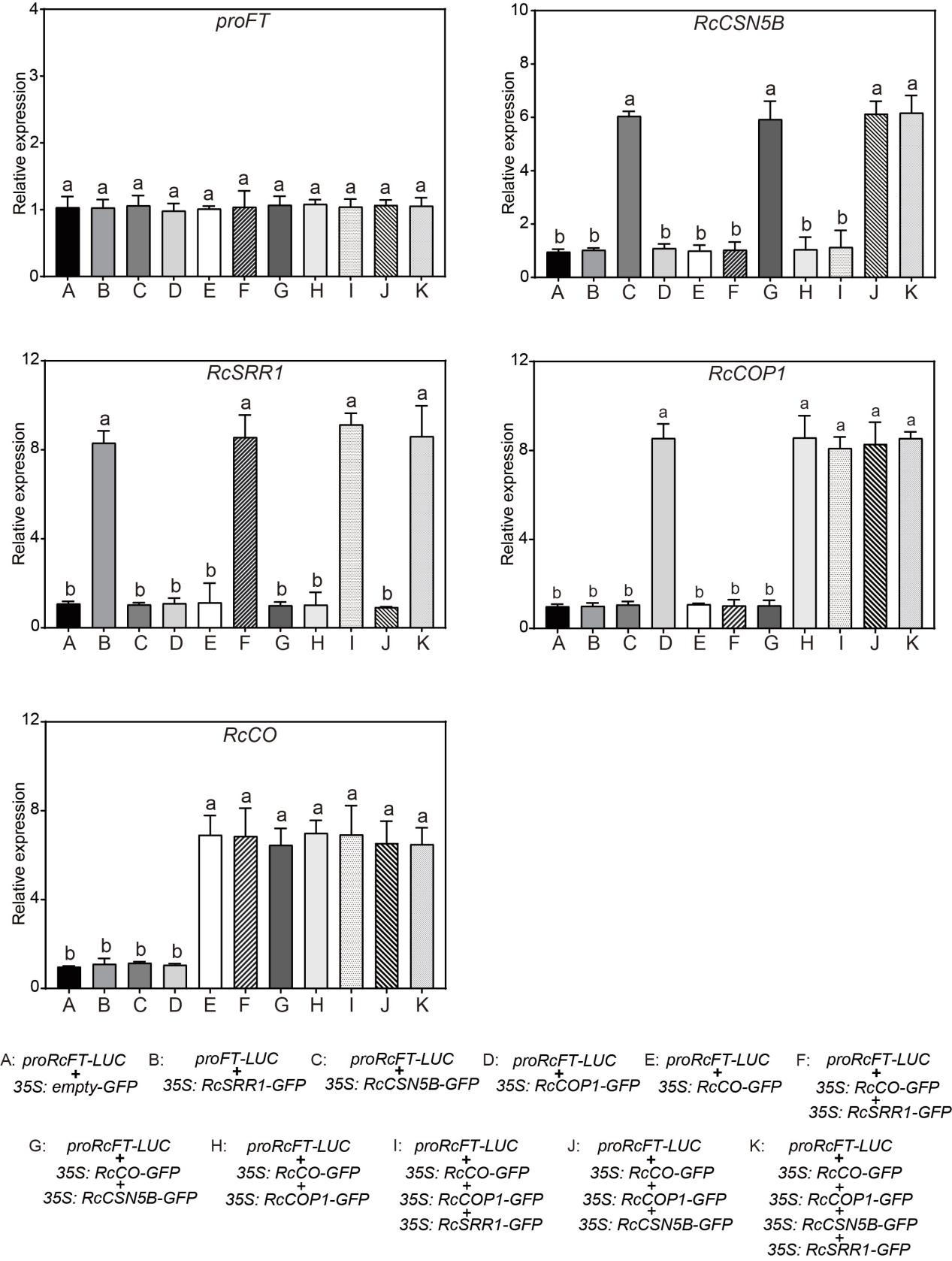


**Supplemental Figure S9 Expression efficiency of individual construct in each treatment of Figure 6B**.

All the relative expression levels were determined by RT-qPCR with *RcGAPDH* as a reference gene. Mean value ± standard deviation was shown from 3 biological replications (n=3) each with 3 technical replications. The different letters meant significant differences at *P*<0.05 conducted with one-way ANOVA followed by Tukey’s multiple range test.


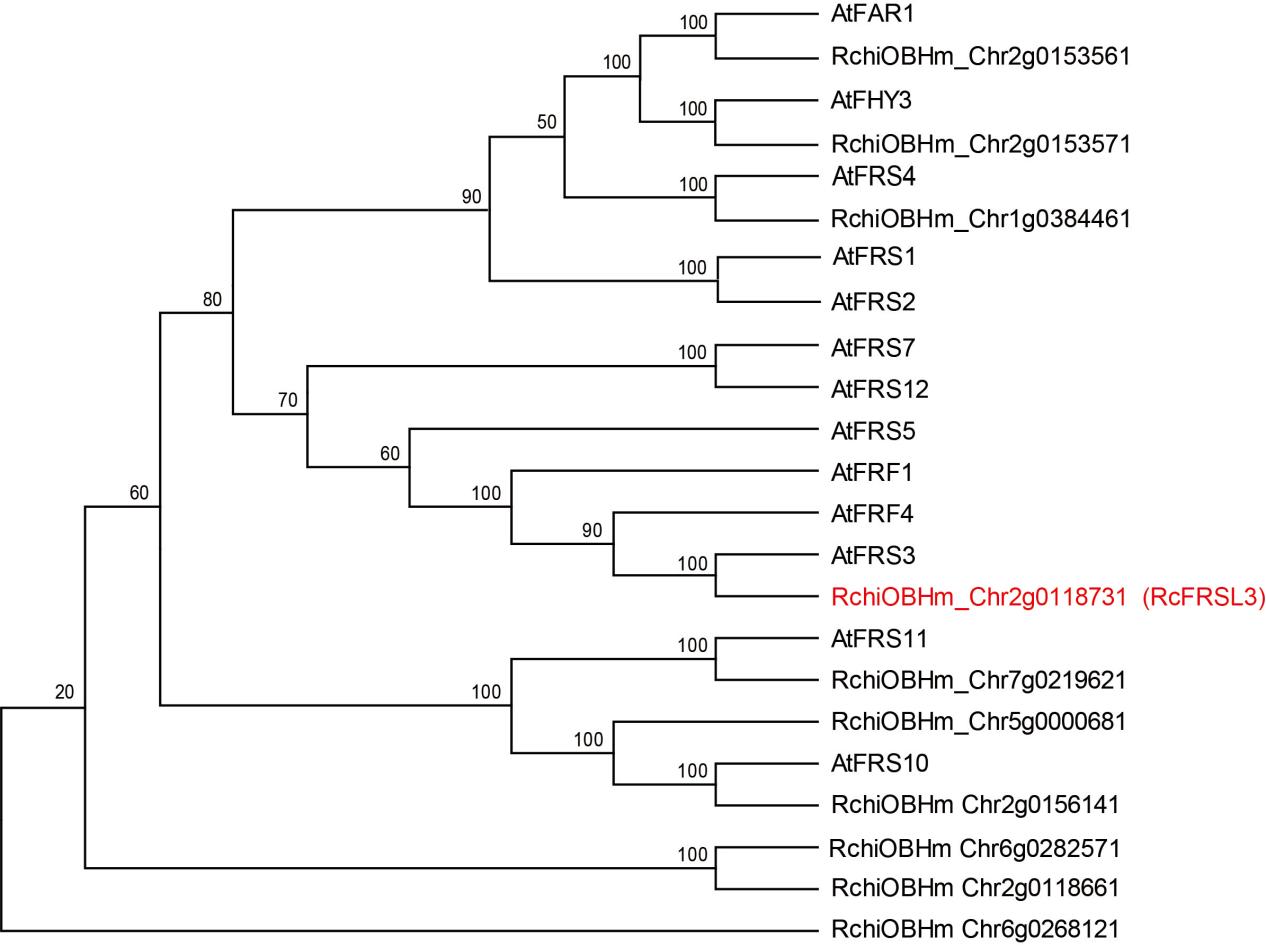


**Supplemental Figure S10 Phylogenetic tree of RcFRSL3 and its homologous proteins in *R*. *chinensis* and *A*. *thaliana.***

The phylogenetic analysis was performed based on the neighbor-joining method. Both the multiple sequences alignment and the phylogenetic tree construction were performed in MEGA version 11.0.13. The values at the nodes represented bootstrap values from 1000 replicates.


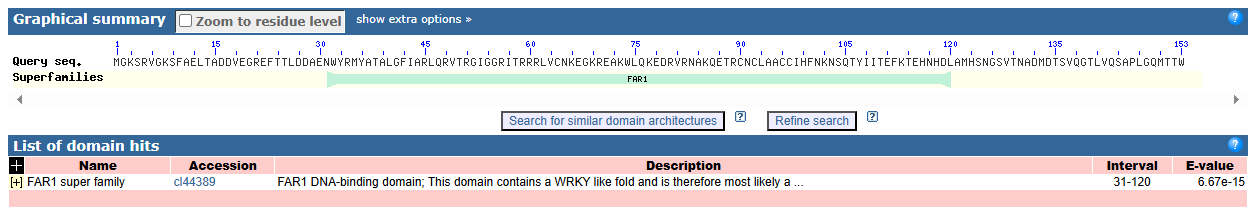


**Supplemental Figure S11 Conseved domain prediction of RcFRSL3**

Conseved domain prediction was obtained by submitting full length amino acid sequence of RcFRSL3 to NCBI website (https://www.ncbi.nlm.nih.gov/Structure/cdd/wrpsb.cgi).


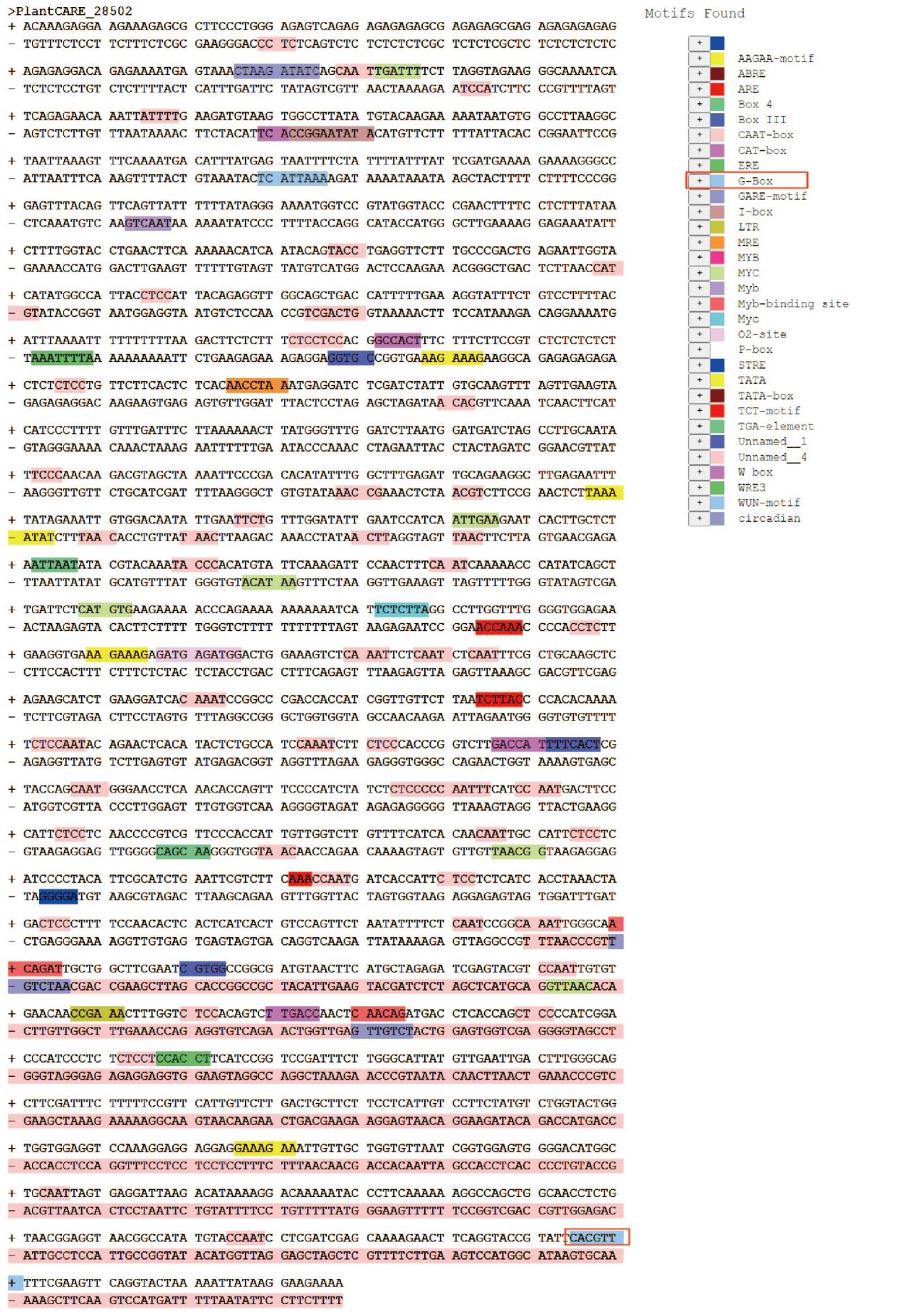


**Supplemental Figure S12 Cis-acting element analysis of *RcSRR1* promoter**

The cis-acting element analysis result was obtained by submitting a 2000-bp *RcSRR1* promoter sequence to the PlantCARE website (https://bioinformatics.psb.ugent.be/webtools/plantcare/html/). The G-box within the *RcSRR1* promoter was highlighted with a red box.


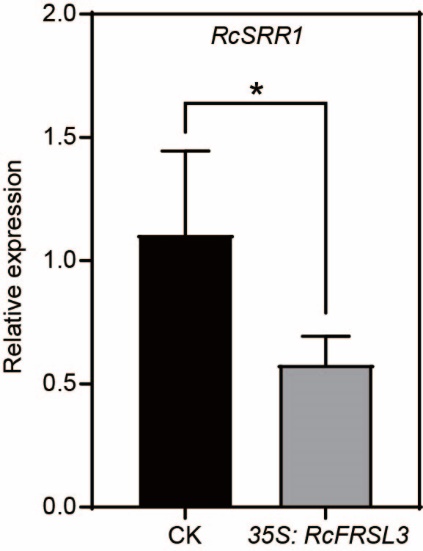


**Supplemental Figure S13 Overexpression of *RcFRSL3* represses *RcSRR1* transcription.**

Mean value ± standard deviation was shown from 3 biological replications (n=3) each with 3 technical replications. The asterisks represented statistically signification differences determined by Student’s *t*-test with **P*<0.05 as the threshold of significance.

Table S1 The potential RcSRR1 interacting proteins identified from yeast two-hybrid assay

| Protein ID | Function prediction | Protein family | Arabidopsis locus |
| --- | --- | --- | --- |
| RchiOBHm_Chr6g0304891 | Rosa chinensis COP9 signalosome complex subunit 5b | MPN | AT1G71230 |
| RchiOBHm_Chr2g0146371 | Rosa chinensis protein TIFY 10b | tify | AT1G72450 |
| RchiOBHm_Chr2g0162081 | Rosa chinensis protein ULTRAPETALA 1 | ULT | AT4G28190 |
| RchiOBHm_Chr7g0213221 | Rosa chinensis MOB kinase activator-like 1B | Mob1_phocein | AT5G45550 |
| RchiOBHm_Chr4g0390271 | Rosa chinensis DNA-directed RNA polymerase V subunit 7 | RNAP_Rpb7_N_like | AT4G14660 |
| RchiOBHm_Chr2g0126111 | Rosa chinensis ribulose bisphosphate carboxylase small chain, chloroplastic | RuBisCO_small_like | AT1G67090 |
| RchiOBHm_Chr4g0427101 | Rosa chinensis S-adenosylmethionine synthase 5 | S-AdoMet_synt | AT3G17390 |
| RchiOBHm_Chr6g0265781 | Rosa chinensis ferredoxin | fer2 | AT2G27510 |
| RchiOBHm_Chr1g0363511 | Rosa chinensis quinone oxidoreductase 1 | QOR2 | AT5G61510 |
| RchiOBHm_Chr3g0472001 | Rosa chinensis enolase | PLN00191 | AT2G36530 |
| RchiOBHm_Chr6g0308781 | Rosa chinensis probable isoaspartyl peptidase/L-asparaginase 2 | Ntn_Asparaginase_2_like | AT3G16150 |
| RchiOBHm_Chr7g0189731 | Rosa chinensis heavy metal-associated isoprenylated plant protein 7 | HMA | AT5G50740 |

Table S2 The potential proteins binding to *RcSRR1* promoter identified

from yeast one-hybrid assay

| Protein ID | Function prediction | Protein family | Arabidopsis locus |
| --- | --- | --- | --- |
| RchiOBHm_Chr2g0118731 | Rosa chinensis protein FAR1-RELATED SEQUENCE 5, transcript variant X2 | FAR1 | AT2G27110 |
| RchiOBHm_Chr5g0063821 | Rosa chinensis ubiquitin-conjugating enzyme E2 7 | UBCc | AT5G59300 |
| RchiOBHm_Chr1g0361101 | Rosa chinensis pyruvate dehydrogenase E1 component subunit alpha-3 | TPP | AT1G01090 |
| RchiOBHm_Chr7g0211981 | Rosa chinensis eukaryotic translation initiation factor 3 subunit J-A | eIF3 | AT5G37475 |
| RchiOBHm_Chr1g0329361 | Rosa chinensis nascent polypeptide-associated complex subunit alpha-like protein 1 | NAC | AT5G13850 |
| RchiOBHm_Chr1g0361101 | Rosa chinensis eukaryotic translation initiation factor 3 subunit M | PCI | AT5G15610 |
| RchiOBHm_Chr1g0362951 | Rosa chinensis probable acetyltransferase NATA1-like | GNAT | AT2G39020 |
| RchiOBHm_Chr4g0443881 | Rosa chinensis 2-methylene-furan-3-one reductase | MDR_like_2 | AT1G23740 |
| RchiOBHm_Chr3g0478671 | Rosa chinensis serine/arginine-rich SC35-like splicing factor SCL33 | RRM | AT1G55310 |
| RchiOBHm_Chr1g0371511 | Rosa chinensis transcription factor SCREAM2 | bHLH | AT2G40435 |
| RchiOBHm_Chr5g0031591 | Rosa chinensis sodium/calcium exchanger NCL | Na_Ca_ex | AT1G53210 |
| RchiOBHm_Chr2g0104571 | Rosa chinensis probable serine/threonine-protein kinase CST | PKc-like | AT4G35600 |
| RchiOBHm_Chr3g0454141 | Rosa chinensis glycine-rich RNA-binding protein RZ1A | RRM2_NsCP33_like | AT3G26420 |
| RchiOBHm_Chr2g0176371 | Rosa chinensis mitochondrial phosphate carrier protein 3, mitochondrial | Mito_carr | AT5G14040 |
| RchiOBHm_Chr7g0194661 | Rosa chinensis UDP-glucose 4-epimerase GEPI48 | GalE | AT4G10960 |
| RchiOBHm_Chr1g0368871 | Rosa chinensis rop guanine nucleotide exchange factor 5 | KPP-like | AT5G05940 |
| RchiOBHm_Chr2g0157181 | Rosa chinensis serine/arginine-rich splicing factor RSZ21 | RRM | AT1G23860 |
| RchiOBHm_Chr2g0163061 | Rosa chinensis 14-3-3-like protein A | 14-3-3 super family | AT3G02520 |
| RchiOBHm_Chr6g0311451 | Rosa chinensis 17.9 kDa class II heat shock protein | HSPs | AT5G12020 |
| RchiOBHm_Chr1g0377051 | Rosa chinensis membrane steroid-binding protein 2 | Cyt-b5 | AT3G48890 |
| RchiOBHm_Chr1g0365321 | Rosa chinensis 60S ribosomal protein L35 | L29 | AT5G02610 |
| RchiOBHm_Chr6g0245911 | Rosa chinensis 60S ribosomal protein L7a-2 | Gadd45 | AT2G47610 |

Supplemental Table S3 Sequences of primers and probes used in this study

| **Assay** | **Gene name** | **Primer name** | **Primer sequence** |
| --- | --- | --- | --- |
| Gene cloning | *RcSRR1* | *RcSRR1*-F | ATGGCATCTTCTGCAAAGAC |
|  |  | *RcSRR1*-R | GCAATTGCTCAACTGCATCT |
|  | *RcCSN5B* | *RcCSN5B*-F | ATGCTTTACCCTTTCTGGGC |
|  |  | *RcCSN5B*-R | ACTTTCAATCATTGGTTCAG |
|  | *RcCO* | *RcCO*-F | CACCATGTTGAAAGAAGAGAGCAATG |
|  |  | *RcCO*-R | GTATGAAGGAACAATGCCGTATC |
|  | *RcCOP1* | *RcCOP1*-F | ATGGGTGGAGGACCAATGTT |
|  |  | *RcCOP1*-R | AGCTGCAAGCACAAGAACTT |
|  | *RcCUL4* | *RcCUL4*-F | ATGTCTCACCCCAACAAACG |
|  |  | *RcCUL4*-R | TGCAAGGTAGTTGTAAACTT |
|  | *RcFRSL3* | *RcFRSL3*-F | ATGGGGAAAAGCCGTGT |
|  |  | *RcFRSL3*-R | CCATGTAGTCATCTGCCC |
| RT-qPCR | *RcFT* | *q*-*RcFT*-F | AGCTTGTGAGTTGTGGGTCT |
|  |  | *q*-*RcFT*-R | ATTGGGAACCGCCCAAGAAA |
|  | *RcCO* | *q*-*RcCO*-F | GATACCACCGAGGACGGGTT |
|  |  | *q*-*RcCO*-R | CAGGACGCAGCCTCATCTTC |
|  | *RcGAPDH* | *q*-*RcGAPDH*-F | GCTGGCAGGTATCCTTTCTG |
|  |  | *q*-*RcGAPDH*-R | GGCGACAATATCAGCCAAGT |
|  | *RcSRR1* | *q*-*RcSRR1*-F | GGATGCTCTTGGTTGCTCTG |
|  |  | *q*-*RcSRR1*-R | GCCTCACAATGTGGCATGAA |
|  | *RcPHYB* | *q*-*RcPHYB*-F | CCATGGTTGTCATTCGCAGT |
|  |  | *q*-*RcPHYB*-R | GAGGAAACGGAATGCACCTC |
|  | *RcGI* | *q*-*RcGI*-F | TGGCCGAAGCAACAATTGAA |
|  |  | *q*-*RcGI*-R | TGCCGAGGAGCTTAAAGGAA |
|  | *RcPIF3* | *q*-*RcPIF3*-F | TTCAGGTGCCTCCTATGCAA |
|  |  | *q*-*RcPIF3*-R | GACCAGGAAGCCCAAACATC |
|  | *RcPIF4* | *q*-*RcPIF4*-F | GCCATGCCTTCTATGCACAA |
|  |  | *q*-*RcPIF4*-R | TGTTGGTGGAGTCACAGTCA |
|  | *RcELF3* | *q*-*RcELF3*-F | CTTGTGGAGGATGTGGACCT |
|  |  | *q*-*RcELF3*-R | TGAGCTTGACATGGTGGGAT |
|  | *RcFRSL3* | *q*-*RcFRSL3*-F | TGCAGAATTGACTGCGGATG |
|  |  | *q*-*RcFRSL3*-R | CAAACCAATCTTCGCCTCGT |
| VIGS | *RcSRR1-*VIGS | *TRV*-*RcSRR1*-F | CCAGGTACATTAAATGGAGA |
|  |  | *TRV*-*RcSRR1*-R | ACACCTCTATATCTCCAATC |
|  | *RcFRSL3-*VIGS | *TRV*-*RcFRSL3*-F | AAACAAGGTTTTTCTCCCAAC |
|  |  | *TRV*-*RcFRSL3*-R | ATGTTTTTGCGTTTCAGATTC |
| Luciferase activity assay | *RcFT* promoter | *proRcFT*-F | ATATCAGTTCTTCATGGCAATCAG |
|  |  | *proRcFT*-R | TAACTAATTTTACACAGGCCACCT |
|  | *RcSRR1* promoter | *proRcSRR1*-F | GACAAAGAGGAAGAAAGAGC |
|  |  | *proRcSRR1*-R | TTTTTCTTCCTTATAATTTT |
| EMSA | Biotin probe | *proRcSRR1*-*Gbox*-F-3' Biotin | TTCAGGTACCGTATTCACGTTTTTCGAAGTTCAGGTACTA |
|  |  | *proRcSRR1*-*Gbox*-R-3' Biotin | TAGTACCTGAACTTCGAAAAACGTGAATACGGTACCTGAA |
|  | Competitor probe | *proRcSRR1*-*Gbox*-F | TTCAGGTACCGTATTCACGTTTTTCGAAGTTCAGGTACTA |
|  |  | *proRcSRR1*-*Gbox*-R | TAGTACCTGAACTTCGAAAAACGTGAATACGGTACCTGAA |
|  | Mutated probe | *proRcSRR1*-m*Gbox*-F-3' Biotin | TTCAGGTACCGTATTAAAGGGTTTCGAAGTTCAGGTACTA |
|  |  | *proRcSRR1*-m*Gbox*-R-3' Biotin | TAGTACCTGAACTTCGAAACCCTTTAATACGGTACCTGAA |
| Y1H | *proRcSRR1*-pHIS2 | *proRcSRR1*-pHIS2-F | TCGGTGGAGTGGGGACATGG |
|  |  | *proRcSRR1*-pHIS2-R | TTTTTCTTCCTTATAATTTT |
| ChIP | P1 | *proRcSRR1*-ChIP-F1 | TGAGGTTCTTTGCCCGACTG |
|  |  | *proRcSRR1*-ChIP-R1 | GGCCGTGGAGGAGAAAGAGA |
|  | P2 | *proRcSRR1*-ChIP-F2 | ATCCGGCAAATTGGGCAACA |
|  |  | *proRcSRR1*-ChIP-R2 | GACCGGATGAAGGTGGAGGA |
|  | P3 | *proRcSRR1*-ChIP-F3 | ACTGGTGGTGGAGGTCCAAA |
|  |  | *proRcSRR1*-ChIP-R3 | TTTAGTACCTGAACTTCGAA |
